# Fully textile passive wireless sensing for human movement monitoring with multiple sensors

**DOI:** 10.3389/fbioe.2026.1724364

**Published:** 2026-02-25

**Authors:** Valeria Galli, Chakaveh Ahmadizadeh, Carlo Menon

**Affiliations:** Biomedical and Mobile Health Technology Group, Department of Health Sciences and Technology, ETH Zurich, Zurich, Switzerland

**Keywords:** activity classification, e-textiles, movement monitoring, multiplexing, wearable strain sensors, wireless systems

## Abstract

Movement monitoring with wearable technologies is becoming increasingly popular in different fields of application (clinical, sports, entertainment). Particularly, textile-based wearables for movement monitoring are attractive as they follow the body movement, are comfortable to use, and can provide continuous tracking capabilities. Ideally, these wearable devices should be flexible (as opposed to current technologies with rigid electronics on the garments) and transmit data wirelessly to avoid hindering the natural movement with connections. Although fully textile wireless and passive wearable systems — whereby the textile sensing part does not have any rigid components and the data is wirelessly transmitted to an external reader — have been developed, the capability of these technologies is currently limited to a single sensor. In this work, we present a system based on a resonating inductor-capacitor (LC) circuits that can measure multiple sensors to broaden the range of use by tracking more than a single joint. Importantly, the presented system employs multiple capacitive strain sensors but retains the use of a single inductor for data transmission, limiting the complexity of realization and the number of connections. After characterization on the bench for careful design of the circuit components, we demonstrated the capability of the system to be used for human movement monitoring and activity classification by integrating two sensors in sport leggings and performing different static and dynamic activities. The tests with sensorized leggings were performed by a single participant. Among a set of chosen classification algorithms, the best performance (F1-score) was 0.98 for the static activities and 0.96 for dynamic activities. When including three independent sessions (donning and doffing the sensorised leggings) accuracy and F1-score dropped to 0.86 and 0.87 respectively. Overall, the presented system has the potential to be adopted as unobtrusive and comfortable smart clothing for real time movement monitoring.

## 1 Introduction

There is an increased interest in monitoring human movement for several applications, and the developments in wearable technologies opened new avenues for real-time continuous motion capture in unconstrained settings. In sports, applications include–but are not limited to–athletic performance tracking (Seshadri et al., 2019), injury prevention (Rebelo et al., 2023), and monitoring the workload of the athlete (Seshadri et al., 2019). In clinical settings, monitoring movement disorders for diagnostic purposes (Ricotti et al., 2023; Schalkamp et al., 2023) or aiding physical rehabilitation (De Fazio et al., 2023) have been explored. Across fields of applications, gait monitoring and biomechanical parameters tracking are one of the main focuses for wearable (strain) sensors as such technology can provide real time continuous monitoring in a user-friendly fashion as compared to traditional, more cumbersome approaches (Shi et al., 2024).

The available technologies for human movement monitoring are either camera-based systems or wearable systems. Camera-based systems range from highly accurate and costly laboratory grade marker-based optical motion capture (OMC) systems–to more recent marker-less portable systems based on fewer cameras (Scataglini et al., 2024). Laboratory grade OMC systems are the accepted gold standard to date but are limited by spatial constraint and long set up times (positioning the markers); mobile OMC systems are less accurate and heavily rely on appropriate camera placement and calibration (El-Rajab et al., 2025). On the other hand, wearable solutions have the clear advantage of following the user’s movement closely and independently of the environment. Inertial measurement units (IMUs) have been largely explored for human motion capture (García-de-Villa et al., 2023), but bear several limitations such as sensor drift and discomfort to the user due to the rigidity of the sensor. Conversely, the use of e-textiles–integrating textile-based sensors in garments–is gaining popularity due to their unobtrusiveness and comfort for the user (Yang et al., 2024).

Various textile-based motion capture systems have been proposed in the literature, with many examples featuring simple demonstrations of the capability of the sensors to track different motions. With specific reference to activity classification or joint position/angle monitoring, several studies focused on upped body pose estimation, using capacitive sensors in loose fitting garments (Bello et al., 2021; Zhou et al., 2023; Yu et al., 2024). Capacitive sensors have also been used for hand gesture recognition in the form factor of a touchpad (Zheng et al., 2018), or integrated in a tight neck collar for head movements and respiration modes classification (Cheng et al., 2010). Regarding the sensing principle, many studies with capacitive sensors have leveraged the capacitance of the human body using it as one of the two electrodes of parallel plate capacitors, with the other electrodes being conductive textile patches (Cheng et al., 2010; Zhou et al., 2023; Kasap et al., 2025) or conductive yarns embedded in seams (Yu et al., 2024). We have previously explored the use of capacitive plates coupled to the body for knee angle estimation using a tight knee sleeve and a random forest algorithm (Galli et al., 2023). Particularly for loose-fitting garments, deep learning and neural network approaches have relied on large-scale datasets (involving multiple participants and extended recording durations) to account for the high variability inherent in capacitive sensing signals that depend on both garment motion and the conductive properties of the body. Parallel plate capacitive pressure sensors have also been employed in socks to classify human gait phases employing neural networks (Vu and Kim, 2020) or as strain sensors in a tight-fitting shirt for shoulder joint angles estimation (Jin et al., 2020).

In addition to capacitive sensing, resistive sensing has also been explored for movement tracking: for example a single strain sensor on the leg was used to classify walking, running and jumping (Vu and Kim, 2018), whereas others combined a screen printed tight-fitting t-shirt with a commercial smart sock for simple activity recognition like sitting, standing, lying down and different walking and running speeds (Mokhlespour Esfahani and Nussbaum, 2019). Other examples of activity recognition with resistive sensors are based on pressure, used for upper body activity or posture recognition in the form factor of arm sleeves (Xu et al., 2022) or shirts (Ma et al., 2024), or gait phase classification with tight leggings (Milovic et al., 2022). A knee sleeve with a combination of pressure sensors and IMUs was also used for lower body joint angles prediction with a Long-Term-Short-Memory algorithm (Zhang et al., 2023).

Supplementary Table S1 reports an overview of the above-mentioned textile-based systems for activity classification with corresponding details on the sensing principles, number of sensors and performance.

From the overview it can be observed that the majority of aforementioned studies have focused on upper body poses, with studies dedicated to lower body often focusing only on gait (Vu and Kim, 2020; Milovic et al., 2022) or running (Tavassolian et al., 2020), or limited to very few activities (Vu and Kim, 2018; Galli et al., 2023). The limited studies investigating more activities of the lower body employ several sensors (Kasap et al., 2025), also with sensor fusion with both textile sensors and IMUs (Zhang et al., 2023).

Although the major contribution of our work is the use of a completely passive and wireless textile multi-sensor system for movement monitoring, we have also explored movement classification in lower body activities with a minimalistic system featuring only two sensors and simple machine learning models. A major differentiation of our work from the above-mentioned state of the art is the absence of rigid electronics on the smart garment: all these examples rely on rigid electronics to collect and transmit data and power: even where sensors are implemented in a textile form, power supply and data communication are provided with rigid or flexible printed circuit boards (PCB). This factor introduces the issue of fragile connections between soft and flexible textiles sensors and rigid electronic modules (Lin et al., 2020; Du et al., 2022). The presence of rigid modules can also hinder natural movement of the user and reduce comfort and breathability, as well as complicate washability since the electronic module needs to be either removable or encapsulated to protect it from moisture.

Removing the rigid parts from the garments and transmitting data and power wirelessly allows to build unobtrusive systems with seamless integration of the sensing components in the garments. In this direction, several advances have been made in the development of wireless, textile-based wearable systems. Some have used clothing to interconnect networks of sensors, however they rely on common wireless protocol like near field communication (NFC) (Lin et al., 2020; Lin et al., 2022) or Bluetooth (Tian et al., 2019) that require rigid components. Critically, the use of passive LC resonance sensing has allowed to eliminate rigid electronics by relying on inductive coupling for wireless communication: previous works have leveraged this well-known principle and developed fully textile systems to track pressure (Wu et al., 2019), humidity (Ma et al., 2019), or human movement (Galli et al., 2023). All these systems were, however, limited to a single sensor, restricting the applicability in the context of human movement monitoring, where complex movements would typically require more sensors. There have been efforts to passively and wirelessly measure multiple sensors (Lee et al., 2025), however also increasing the number of inductors needed and the overall system complexity. Most of these have only shown basic functionality of the sensors to track simple movements and conventionally use one antenna per sensor (even when using more than one sensor): this inevitably increases the system complexity, especially in more sophisticated applications that require multiple sensors such as tracking a joint in more than one plane or tracking complex movements.

Therefore, we have expanded our previously developed textile sensing system for continuous human movement tracking that used only one sensor (Galli et al., 2023) to operate with multiple sensors and still use a single textile antenna and a single external reader with inductive coupling. Multi-resonant sensing circuits in the form of stacked LC resonant circuits have been implemented on flexible substrates for wearable applications (Lee et al., 2020) or in textile integrated systems (Lee et al., 2025). However, these require one inductor for each capacitive sensor, which complicates the design and size of the system. Exploiting a single inductor to connect multiple sensors is advantageous and eliminates the problem of mutual interference among stacked resonant circuits. We have developed this concept by tapping the inductor along its length to connect multiple capacitive sensors: by tuning the inductor and capacitors values, multiple resonant peaks can be detected by an external reader inductively coupled to the inductor. Very few implementations of this concept have been reported for dual parameter monitoring in industrial applications: a rigid humidity and temperature sensor (Dong et al., 2019), or a temperature and strain sensor (Wang et al., 2020) were demonstrated in generally in highly controlled conditions. However, there is no example of a wearable textile system featuring passive wireless data readout of multiple sensors from a single readout point using a single textile inductor (antenna) capable of tracking real time human movement data.

**FIGURE 1:**
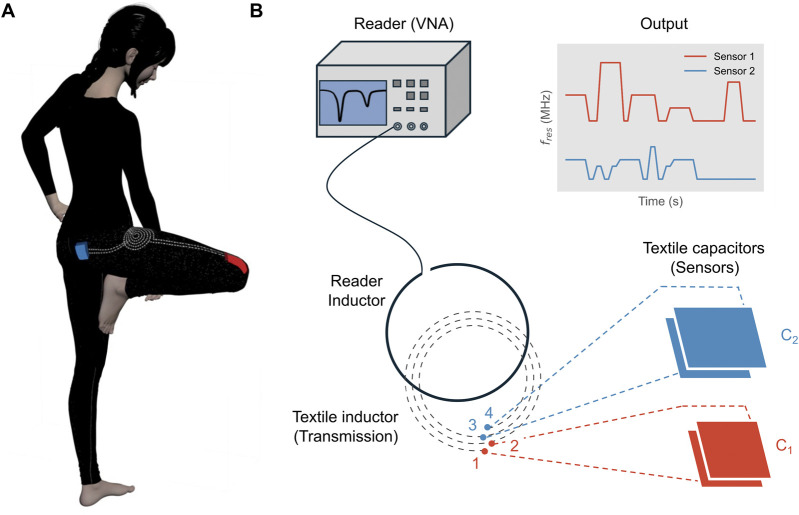
Overview of system. **(A)** Sensorised garment (sport leggings) with multiple textile capacitive sensors connected to a single textile inductor; **(B)** The textile inductor is interfaced with an external reader (VNA) via inductive coupling with a reader inductor and the resonant peaks (*f*_*res*_) can be identified from the reflection coefficient *S*_*11*_. Each sensor’s stretching state changes during the activities and their corresponding resonance frequencies can be monitored in time (output).

Figure 1 shows the overall concept: the garment is equipped with multiple textile capacitive strain sensors in various locations, all connected to a single textile inductor (Figure 1A). An external reader inductor is coupled with the textile inductor to transmit the data from the sensor network. The reader inductor is connected to a vector network analyzer (VNA, reader device) and the resonance frequency of each sensor is detected as a dip in the reflection coefficient (*S*_*11*_) signal (Figure 1B). Each sensor taps the inductor at a different location (Figure 1B), allowing multiple resonant peaks to be detected without mutual interference. To our knowledge, this is the first wearable textile system featuring fully passive, wireless readout of multiple sensors for real-time human movement monitoring. Importantly, this architecture enables all sensors to be read wirelessly through a single readout point, eliminating the need for multiple antennas, wiring routes, or dedicated readout units. This significantly reduces system complexity, improves wearability and facilitates scalable integration of additional sensors without increasing readout hardware. A single participant performed 10 different activities in three independent sessions (doffing and donning the sensorised leggings, on three different days). The proposed design can be adapted to different body locations and demonstrates the feasibility of tracking and classifying common human activities using the proposed technology. The main contributions of this work are:

- Development of a fully passive, all-textile wearable system integrating multiple capacitive strain sensors.
- Wireless data and power transmission *via* inductive coupling from a single readout point.
- Feasibility demonstration for activity monitoring of common human movements.
- Flexible and adaptable design suitable for unobtrusive, real-time continuous movement tracking.

## 2 Materials and methods

The main requirement for the proper functioning of the system is to distinguish multiple resonant peaks (one for each sensor) and their corresponding shift when stretching the sensors. In practical terms, this requires sufficiently distant resonance frequencies for each sub circuit. For each subcircuit, the resonance frequency can be approximated by the theoretical formula:

$$f_{i}=\frac{1}{2\pi \;\sqrt{L_{i}C_{i}}}$$ (1)

Where $L_{i}$ is the inductance of each sub-inductor and $C_{i}$ the capacitance of each sensor, provided that the resonances are distinguishable (i.e., not overlapping). Therefore, the system can be tuned by using appropriate values for $L_{i}$ and $C_{i}$ such that $f_{1}<f_{2}<\ldots <f_{i}$ (Figure 1B) when the sensors are at rest or being stretched. This translates into designing a textile inductor with a relatively high self-resonance (i.e., behaving as inductor up to self-resonance), and capacitive sensors with capacitance at unstretched and stretched states different enough to generate resonance frequency peaks apart in the frequency spectrum that can be clearly recognized. The most straightforward way to ensure good distinction between the peaks was to tune the values of the capacitive sensors by tuning their geometry. Based on Equation 1, when adding more sensors to the system (i.e., instead of a single sensor *C*_*1*_), decreasing the baseline capacitance for the added sensors (i.e., $C_{1}>C_{2}>\ldots >C_{i}$ ) would result in progressively higher resonance frequency (i.e., $f_{1}<f_{2}<\ldots <f_{i}$ ). Moreover, connecting the largest capacitor to outermost turn of the inductor (sub-inductor $L_{1}$ ) which also has the largest inductance (larger enclosed area) would accentuate this effect ( $L_{1}>L_{2}>\ldots >L_{i}$ ).

### 2.1 System components design and characterization

#### 2.1.1 Textile inductor

For the textile inductor, conductive yarn was embroidered in a circular spiral geometry onto standard spandex substrate. The inductance that can be obtained with embroidered textile inductors is limited by the conductivity of the utilized conductive yarns–typically lower than copper wire and copper traces on PCBs–and by the size. To obtain a small inductor that can fit in the pocket of a garment, an inductance of a few microhenries can be obtained. Geometrical parameters like the overall enclosed area, the number of turns and the gap between each turn determine the overall inductance, as described by several formulas in literature [Wheeler’s formulas and modifications thereof (Mohan et al., 1999)].

A circular shape was chosen over square or rectangular shapes to minimize the stress on the yarn during embroidery by avoiding sharp edges and because such shape has been shown to be less influenced by in-plane rotational misalignment with the reader coil in inductive coupling (Grabham et al., 2018). Also, it was previously shown that circular spiral inductors have similar performance to square inductors in inductive coupling (Heo et al., 2018) and are more tolerant to in-plane misalignment with the reader inductor. The final geometry for the textile inductor was chosen based on the following criteria:

- compact size to be placed in garment pocket;
- high self-resonance to allow for a large enough frequency range where multiple resonance frequencies (one per subcircuit) could be detected.

The textile inductor was embroidered with a programmable embroidery machine (Innov-is N26000, Brother, US) with a silver plated Vectran Liberator 40-Ag yarn [Syscom Advanced Materials, US (Liberator conductive fibers, 2022)]. Despite being stiffer and more difficult to embroider compared to other commercially available conductive yarns on the market, this yarn was chosen for its high conductivity (3.3 Ω/m) that is essential in wireless circuits applications.

For optimal wireless power and/or data transmission, it is generally desirable to have a high quality factor of the inductors (Lee et al., 2021; Lin et al., 2022). A low quality factor results in broad *S*_*11*_ peaks and therefore complicates the identification of the resonance frequency from the peak minima. Especially when looking at frequency shift for sensing purposes (as in this case where the shift is induced by strain on the capacitive sensors), if the *S*_*11*_ peaks are not sharp (low quality factor) small shifts can be difficult to detect. Quality factor is defined as:

$$Q=\frac{1}{R}\;\sqrt{\frac{L}{C}}$$ (2)

Where *R* is the equivalent series resistance, *L* is the inductance, and *C* is the parasitic capacitance of the inductor. The quality factor is inversely proportional to the overall resistance of the circuit, raising the need for the highest possible conductivity in the yarn used for the inductor and the connections. For a spiral inductor, inductance can be approximated by the following equation–the modification of the simplified Wheeler’s formula for circular planar spiral inductors (Shah et al., 2014):

$$L=\frac{1}{4}\;\mu_{0}\;N^{2}\;\left(d_{in}+d_{out}\right)\left[\ln\frac{2.46}{p}+0.20\;p^{2}\right]$$ (3)

Where $\mu_{0}$ is the permeability of free space (4π × 10^−7^ H/m), $d_{in}$ is the inner diameter of the coil, $d_{out}$ the outer diameter, N the number of turns, and *p* is the fill factor, which is defined as:

$$p=\frac{d_{out}-d_{in}}{d_{out}+d_{in}}$$ (4)

The inner and outer diameters are related to each other by the number of turns *N*, the width of the trace *w* (for embroidered inductors, the width of the utilized conductive yarn) and the spacing (gap) between the turns *s*:

$$d_{in}=d_{out}-\left(2N+1\right)\;w-\left(2N-1\right)\;s$$ (5)

To obtain a high quality factor *Q* for the inductor by increasing *L*, the outer diameter can be increased, and the gap can be decreased. For the practical purpose of placing the inductor in the pocket of a garment, the maximum outer diameter $d_{out}$ was set to 40 mm. As for the spacing between turns (gap *s*), the lower limit was given by the yarn diameter (nominal value 0.23 mm) and by the embroidery technique: to avoid short circuits, a minimum gap of 1 mm was chosen. It is also important to note that increasing the overall size ( $d_{out}$ ) also translated to higher overall length of the inductor and consequently higher equivalent series resistance (*R* in Equation 2). Substituting the values for the diameters and fill factor (Equations 4, 5) in Equation 3, an estimate of the inductance with the geometrical limitations described above can be obtained, with theoretical inductance around 0.5 μ.

The other key factor to consider is the self-resonance of the inductor: as mentioned above, high self-resonance is desirable to allow for a sufficient frequency range below self-resonance where the inductor behaves. A higher inductance or parasitic capacitance would drive self-resonance down (following the formula for resonant circuits, Equation 1). The parasitic capacitance increases with a decrease in the spacing between turns (lower gap *s*).

As for the number of turns, each capacitive sensor was connected to a part of the spiral inductor (sub-inductor) that was itself an inductor, therefore at least one turn was used for each sensor: for example, to use two capacitive sensors, the textile inductor would need to have at least two turns. In addition, to facilitate the application of connections, an additional turn could be left between each pair of sensors (Figure 1B).

Overall, the aim was to have an inductor with sufficient inductance and quality factor for good magnetic coupling, and at the same time high self-resonance. When using two sensors, for example, a self-resonance of 100 MHz would be sufficient to ensure a large enough frequency range for the two resonances (one per sensor) to be far apart (e.g., 15–20 MHz) and clearly distinguishable.

Inductors with a fixed outer diameter of 40 mm, 3 turns and varying gap (1–4 mm) were manufactured (Supplementary Figure S1), and their impedance characteristics were analyzed with an impedance analyzer (HIOKI IM7587, Japan). The effect of bending on inductance was also investigated as the inductor is to be placed on the waistband of tight sport leggings and therefore subject to bending depending on the wearer’s body shape. To obtain the sub-inductors, connections to the desired points were hand sewn with conductive yarn and used to connect the sensors (Supplementary Figure S2). The inductance of each sub-inductor was also probed with the impedance analyzer.

#### 2.1.2 Textile capacitive strain sensors

Capacitive sensors were fabricated with a parallel plate design with conductive spandex (Electrolycra, Mindsets, United Kingdom) and standard spandex as the dielectric layer. The conductive spandex was laser cut to the desired shape (Rayjet, Trotec, Austria) and heat press on the non-conductive spandex *via* a stretchable adhesive web (HeatnBond, ThermOweb, United States).

The baseline capacitance could be tuned by modifying the area of the electrodes and the dielectric layer thickness, based on the formula for parallel plate capacitors:

$$C=\frac{\varepsilon \;A}{d}=\frac{\varepsilon_{0}\;\varepsilon_{r}\;A}{d}$$ (6)

Where *C* denotes the capacitance, *A* the area of the plates, *d* the distance between the plates, and ε the absolute (complex) permittivity of the dielectric layer (product of free space permittivity $\varepsilon_{0}$ and relative permittivity of the material $\varepsilon_{r}$ ). The relative permittivity of textiles has been previously reported to be between 1.5 and 2 in the GHz range (Sankaralingam and Gupta, 2010). We measured the permittivity of the utilized non-conductive spandex with a dielectric probe kit (DAK-12, Speag, Switzerland) connected to the VNA (E5061B ENA, Keysight, US) in the range of interest for our system (MHz).

Substituting the values for the diameters and fill factor (Equations 4, 5) in Equation 3, an estimate of the inductance with the geometrical limitations described above can be obtained, with theoretical inductance around 0.5 microhenries. The capacitive strain sensors with parallel plate design were produced by heat pressing and then stitching conductive stretchable spandex onto non-conductive spandex to ensure good adhesion. The conductive spandex is highly stretchable along its length (up to 100%) and has a surface resistivity of about 1 Ω/sq (ElectroLycra, n. d.). Conductive yarn (Liberator) was stitched on each electrode for connections. In practice, two separate layers (one per electrode) made of conductive spandex heat pressed onto a non-conductive spandex layer. Using such a structure instead of directly applying conductive spandex on either side of a single non-conductive spandex layer was aimed at preventing any electrical short circuits at the locations where conductive yarn connections were stitched to the electrodes. The two layers of non-conductive spandex with one electrode each were then stitched together with electrodes facing outward. Supplementary Figure S3 shows the steps of the sensor fabrication in detail.

The baseline capacitance was tuned by changing the shape and size of the plates. The distance between the electrodes in the non-stretched configuration was assumed to be consistent across capacitors as the same spandex substrate and manufacturing method was applied to all sensors. A single layer of non-conductive spandex was around 0.4 mm thick, therefore a distance of 0.8 mm was assumed for the two layers.

The baseline capacitance and stretchability of the sensors could be tuned by changing the shape and size. Using a larger sensor and a thinner dielectric layer (e.g., minimum amount of insulating fabric layers) would yield a higher capacitance and conversely reducing the area or increasing the number of insulating layers would decrease the baseline capacitance.

Moreover, for rectangular sensors of the same length, thinner sensors (smaller width) are less stiff and therefore stretch more easily than wider ones. (thinner rectangle) allowed for higher compliance along the length. This factor is especially relevant considering the location where sensors are applied on the garment when applying the sensors on garments, as the stretch on the sensor depends on the positioning on the garment. For instance, a sensor placed above the kneecap will undergo extensive stretching during knee flexion, but other areas, such as the glute region, may not experience the same degree of strain. Therefore, the minimum length was set to 60 mm and the maximum width to 15 mm to limit stiffness.

The electromechanical behavior of the capacitive sensors (i.e., the change in capacitance upon application of strain) was assessed by repeatedly stretching the sensors using a universal tensile test machine (UTM) (Zwick-Roell Z1.0, Germany) and simultaneously measuring the sensors impedance with a precision inductance capacitance resistance (LCR) meter (Hioki IM3536, Japan). Supplementary Figure S4 shows one of the sensors mounted on the UTM *via* custom 3D printed fixtures. Custom made 3D printed fixtures were used with the UTM, and the tests were video recorded to track the actual area change of the capacitive sensors’ electrodes (conductive fabric part). This step was necessary as the applied displacement from the UTM would inevitably also stretch the non-conductive spandex at the top and bottom of the electrodes such that the applied displacement did not directly correspond to the actual strain on the electrodes. The area change was calculated by detecting the electrodes’ contour throughout the test with the *OpenCV* library.

To characterize the sensitivity of the sensors, 10 preconditioning cycles were applied at 100% strain followed by cycles of strain with increasing strain values up to 30% (5, 10, 15, 20% and 30%). 10 cycles of stretch-release were repeated at each strain value at a rate of 5 mm/s. The upper limit of 30% was chosen as the maximum strain once the sensors are applied on the garments is expected to remain lower than 30% (Geng et al., 2021). Afterwards, step-hold tests were performed at different levels of strain to observe any static drift and to simulate the application of pose estimation (static activity classification). The sensors were strained to 5%, 10%, 15% and 20% and then back to the initial state following the same values. Each level of strain was held for about 4 s (hold phase). This test also allowed us to compare the different behavior of the sensors when stretched vs. released (hysteresis). Three full cycles of the step-hold test were applied and the resulting response of the sensors averaged across them was used to characterize the sensors.

### 2.2 Simulations

Once the values of inductance for the sub-inductors were determined, appropriate values for capacitive sensors were sought by simulating the circuit and varying the capacitance parametrically to assess whether the corresponding resonance frequencies would be distinguishable. Simulations were performed in *LTspice* (LTspice, n. d.) using AC analysis with a frequency sweep of 10–100 MHz and 200 points per decade. Supplementary Figure S5 shows the schematic for the circuit. The VNA was simulated as an AC source with series resistance of 50 Ω, and a 50 Ω load (typically used in radiofrequency applications with VNAs) was placed in parallel to the circuit to run the network analysis in the simulator. Each component of the circuit was assigned the impedance characteristics measured with the impedance analyzer. Supplementary Table S5 reports the measured impedance for the connection lines. Lastly, the mutual inductance between the reader inductor and each sub-inductor had to be defined for the simulation. The mutual inductance *M* between two inductors (inductive coupling) is defined as:

$$M=k\;\sqrt{L_{R}L_{s}\;}$$ (7)

Where *k* is the coupling coefficient*,* $L_{R}$ is the reader inductor, and $L_{S}$ is a generic textile inductor (any sub-inductor). For circular planar spiral inductors, the coupling coefficient can be approximated with the formula:

$$k\left(d\right)=\frac{1}{{\left[1+2^{\frac{2}{3}}\;{\left(\frac{d}{\sqrt{r_{1}r_{2}}\;}\right)}^{2}\right]}^{\frac{3}{2}}}$$ (8)

Where d is the axial distance between the two inductors and $r_{1}$ and $r_{2}$ are the effective radii of the two inductors calculated using the following formula (arithmetic mean of inner and outer radii):

$$r_{eff}=\frac{r_{in}+r_{out}}{2}$$ (9)

The effective radius for each sub-inductor was calculated, and the corresponding coupling coefficients for axial distance *d* between reader and textile inductor between 1 and 10 mm were similar across sub-inductors (Supplementary Table S6). The effective radii were calculated with Equation 9 and plugged into Equation 8 to estimate the coupling coefficient k. A coupling coefficient of k = 0.35 was chosen considering that an axial distance between reader inductor and textile inductor between 1 and 2 mm, as the two were physically and tightly overlapped, with the spatial constraint of the pocket and compression from the user’s body when wearing the tight-fitting leggings. Therefore, the calculated coupling coefficient for each sub-inductor was approximately k = 0.35 for both L_1_ and L_2_ as reported in Supplementary Table S6. The highest possible coupling coefficient is desired to ensure high mutual inductance (Equation 7) and therefore strong inductive coupling. In the ideal case (no losses), k = 1, whereas in realistic scenarios k is typically around 0.5 or lower.

As regards in-plane misalignment between reader inductor and textile sub-inductors, the design of the tight pocket stitched on top of the textile inductor ensured physical confinement of the reader inductor such that in-plane misalignment was largely prevented. Although we previously demonstrated that a 50% lateral misalignment between the two inductors results in complete signal loss (i.e., no detectable S_11_ peak; Supplementary Figure S7 in Galli et al., 2023), such displacement was not physically possible in the present setup. By contrast, axial misalignment has a more pronounced effect on signal amplitude and quality factor: when the separation between the inductors exceeds approximately 25 mm, no resonance frequency can be detected. In practical application, the pocket design restricted axial displacement to a few millimeters.

The baseline values for the capacitors based on the chosen length and width ranges were first calculated based on Equation 6 (Supplementary Table S4). An increment of 1 pF per step was chosen to show the change in resonance frequency as the sensor would be stretched. Simulations were run for combinations of two sensors (*C*_*1*_ and *C*_*2*_) with the calculated baseline values by varying *C*_*1*_ and *C*_*2*_ parametrically with a step of 0.5 pF. Only combinations with *C*_*1*_ *> C*_*2*_ were considered, following the design principle explained above: to best distinguish resonances such that $f_{1}<f_{2}$ , capacitive sensors were chosen such that $C_{1}>C_{2}$ (Equation 1).

### 2.3 Wireless sensing system response to controlled strain

To characterize the response of the system to strain, bench tests were conducted where multiple sensors were strained first one at a time and then simultaneously.

The sensors were connected to the textile inductor *via* stretchable connection lines. The first sensor was connected to the first turn (first sub-inductor, between points 1 and 2 in Supplementary Figure S2), and second one was connected to the last turn (last sub-inductor, between points 3 and 4 in Supplementary Figure S2), leaving a small inductance in between to better isolate the sub-circuits (*L* in the circuit schematic, Supplementary Figure S5). The reader inductor was a custom-made circular loop made of single stranded wire connected to the VNA with SubMiniature version A (SMA) connector and cable. The outer diameter of the reader inductor matched the outer diameter of the textile inductor (set to 40 mm) for optimal inductive coupling. The reader inductor was connected to a VNA *via* an SMA coaxial cable, and the resonance frequencies deriving from the two sensors were tracked continuously.

As it was not possible to test more than one sensor with the UTM at the same time, custom 3D printed fixtures were used: each sensor could be mounted on the fixture and stretched by a discrete small displacement and be held in place (Supplementary Figure S6). Each capacitive sensor was connected to the corresponding part of the textile inductor, and a reader inductor was coupled with the textile inductor and connected to the VNA to track the resonance frequencies (one per sensor) as depicted in Figure 1B.

Each sensor was stretched and held in place for about 4 s and the corresponding shift in the resonance frequency was identified as the minimum of the *S*_*11*_ reflection coefficient. First, one sensor was stretched at a time to strain levels of 5, 10, 15% and 20%. Then one sensor was stretched at a specific strain level and the other simultaneously stretched to observe any interference among multiple sensors.

### 2.4 Activity classification with sensorised garment

To demonstrate the capability of the system to track different activities, two sensors were integrated into sport leggings, one above the kneecap and one on the glute of the same leg (Supplementary Figure S7). The connection between each sensor and textile inductor was made of conductive yarn in a serpentine shape to ensure stretchability and not hinder the user’s movement during the activities (Supplementary Figure S8). The textile inductor was stitched to the waistband inside a tight pocket, such that the reader inductor could be placed in the pocket and be kept aligned with the textile inductor throughout the activities.

Different static and dynamic activities were performed: squat, deep squat, foot to glute, knee drive, hip flexion, hip abduction, lunge, walking and jogging on the spot (Supplementary Figure S9). A single participant performed all the tests. This study was granted exemption by ethical approval by the ETH Zurich Ethics Commission (exemption determination number EK 2025-E-01). All methods were conducted in accordance with relevant guidelines and regulations, and all experimental protocols were approved by the ETH Zurich Ethics Commission. Written informed consent was obtained from the participant including consent to publish images displaying them upon removal of any identifying information.

Each test started and finished with standing as baseline signal. For static activities, each pose was held for about 4 s followed by 4 s of standing, repeating for 10 cycles. For dynamic activities, poses were repeated continuously for 10 cycles and walking and jogging on spot were repeated for 20 steps each. Both static and dynamic activities were recorded at a sampling frequency of $f_{sampling}=70\;Hz$ (i.e., one frequency sweep saved every 0.014 s). To achieve such sampling rates, the frequency range for the sweep was limited to the minimum range that included the reflection coefficient (*S*_*11*_) peaks of both sensors in the stand and pose phases, and the frequency resolution was set to 200 kHz. For example, for a frequency sweep between 20 MHz and 60 MHz, a resolution of 200 kHz implies 200 sweep points. There is a tradeoff between the attainable sampling rate (especially, a stable sampling rate) and the frequency range and resolution. As the performed activities were either static or had maximum frequency just above 1 Hz for the dynamic tests, $f_{sampling}=70\;Hz$ was largely sufficient to capture the signals features (considering the Nyquist theorem and additional margin). It is noteworthy that none of the commercially available portable VNAs at the time of writing could achieve such sampling rate, therefore a benchtop VNA was chosen for these tests.

From each single per frequency sweep that was recorded, the resonance frequency for each sensor was detected as the minimum in the *S*_*11*_ reflection coefficient: for a system with two sensors, two *S*_*11*_ (negative) peaks were expected and therefore two resonances. As each of the sensors were stretched, its capacitance would increase and therefore the resonance frequency of the corresponding sub-circuit would decrease according to Equation 1. All data was low pass filtered to remove high frequency noise: static tests were filtered with a cutoff frequency of 1 Hz, dynamic tests with cutoff frequency of 5 Hz.

For each test (both static and dynamic), the resonance frequencies of the two sensors were tracked and the phases (“standing” vs. “activity”) automatically labeled based on a threshold approach: for “standing” (sensors unstretched, higher resonance frequency) the threshold was chosen as the maximum resonance frequency minus a tolerance of 20% of the full range:

$$f_{threshold_{stand}}=\max\left(f\right)‐0.20\;\left(\max\left(f\right)‐\min\left(f\right)\right)$$ (10)

Whereby all data points with ${f\left(t\right)\geq f}_{threshold_{stand}}$ were labeled as “standing” phase.

Similarly, the threshold for “activity” (sensors stretched, lower resonance frequency) was chosen as the minimum resonance frequency plus a tolerance of 15% of the full range:

$$f_{threshold_{activity}}=\min\left(f\right)+0.15\;\left(\max\left(f\right)‐\min\left(f\right)\right)$$ (11)

Where all data points with ${f\left(t\right)\leq f}_{threshold_{activity}}$ were labeled as “activity” phase. The tolerances were needed to capture the full cycles as both sensors naturally had fluctuations in the response.

Different tolerances were selected for standing and activity phases as it was observed that the signals for both sensors in the standing phases were noisier due to the adjustment of the sensors in the unstretched state (e.g., the sensor slightly sagging). All datapoints in between standing and activity phases–corresponding to the transitions between the two positions–where labeled as “transition”. If for a specific timestamp the labels for the two sensors were different (most commonly: one labeled as “transition” and one as either “activity” or “standing”) and at least one sensor value identified as standing or activity phase, the “standing” or “activity” label was set for both: this allowed to retain data where one sensor had more noisy data (e.g. some values falling outside the threshold (and therefore being labeled as “transition”) for one sensor but not for the other.

After assigning labels, features were extracted from the signals using overlapping windows (80% overlap) of 1 s for static activities and 0.2 s for dynamic activities. The window sizes were chosen based on a trade-off between the frequency of the performed activity, the number of data points for each phase/cycle, and correct labeling of each window. Preliminary experiments with longer windows for static activities showed that many windows spanned multiple postures (e.g., part standing, part squatting), which led to incorrect labeling of some windows. Shortening the window to 1 s, combined with 80% overlap, resolved this issue by aligning windows mostly within steady-state portions of each posture, preserving the correct labels for each window and generating more training samples for robust feature extraction. For dynamic activities, shorter windows (0.2 s) were required for similar reasons: longer windows would capture multiple movement phases, causing label confusion and the need for manual adjustment.

The following features were initially explored: mean, standard deviation, root mean square (RMS), mean of derivative, standard deviation of derivative, range, slope, entropy, dominant frequency, skews, kurtosis, and correlation between the two sensors signals. Feature importance was evaluated with Anova F-score and mutual information.

Features were normalized with zero mean and unit standard deviation.

The classification task was performed on the static and dynamic datasets separately. All the activities in the static tests were also included in the dynamic tests, therefore classification on the dynamic tests would be a representative result for the whole dataset.

For both datasets, the data was split into training-validation-testing set (80%–10%) based on cycles to preserve temporal structure of the data: for most activities, 10 consecutive cycles were performed, eight cycles were included in the training set, one cycle in the validation set, and one in the testing set. For walking and running, 20 steps (equivalent to 20 cycles of activity) were recorded, therefore the splitting was 16 cycles for training, two cycles for validation, and two cycles for testing. Standard machine learning classification models were applied: K-Nearest Neighbors (KNN), Logistic Regression, Support Vector Machine (SVC), Decision Trees, Random Forest (RF), and Extreme Gradient Boosting (XGB).

Hyperparameter tuning and 4-fold cross validation were performed on the training and validation sets, and the trained models were used for prediction on unseen test data separately, to avoid any data leakage. The hyperparameter space used for each model is reported in Supplementary Table S8. Accuracy and weighted F1-score were used as metrics to evaluate each model’s performance on the test set.

In a subsequent analysis, the same protocol for dynamic activities (no hold phases) was repeated by the same participant on two separate days with the same prototype (sensorised leggings), obtaining three independent sessions. For this analysis, the same approach was use (same features, same scaling and same hyperparameter tuning), with the addition of two classification models: Light Gradient Boosting Machine (LGBM) and Categorical Boosting (CatBoost) that have shown potential for human activity recognition from wearable sensors (Lalwani et al. 2025; Liu and Chen, 2025).

All the machine learning analysis was conducted in Python 3 using the *scikit-learn* library.

## 3 Results and discussion

### 3.1 System components

#### 3.1.1 Textile inductor

For the textile inductor, all four inductors with gaps between turns of 1, 2, 3, and 4 mm showed a self-resonance above 100 MHz (Supplementary Figure S10). The average inductance below self-resonance ( $f<\frac{f_{self_{res}}}{2}$ ) was calculated using the recorded values of inductance in the range $f<f_{self_{res}}$ (inset to Supplementary Figure S10). As expected, inductance *L* increased with decreasing spacing between turns (Supplementary Table S2). The measured inductance was higher than the one calculated from Equation 3 for all designs due to the added inductance of the connections at each end of the inductors. Such connections were kept as short as possible but were needed to probe the inductor with the impedance analyzer fixture. For all designs, the same connection length of 5 cm was used.

The highest inductance is desirable for stronger magnetic coupling, i.e., the inductor with the smallest gap, *s = 1 mm*. However, tapping the inductor with such a small gap between turns–i.e., stitching connections to the capacitive sensors on each turn–proved challenging as the tapping points could create short circuits between turns. Therefore, the inductor with *s = 2 mm* was chosen.

For this design, the impedance of the 3 sub-inductors was observed to decrease progressively from the outermost (first sub-inductor *L*_*1*_) to the innermost (third sub-inductor *L*_*3*_) as expected since the enclosed area and overall length of the sub-inductor decrease from the first (outer) turn to the third (inner) turn (Supplementary Table S3). All sub-inductors had a self-resonance above 200 MHz, which was high enough to form resonant sub-circuit with the corresponding capacitive sensors in a frequency range well below self-resonance.

The inductor was stitched in a taut configuration in a pocket on the waistband of the tight-fitting leggings, where minimal movement and essentially no stretching was present. Considering the small size of the inductor (40 mm diameter) and the chosen position, very deformation of the inductor was observed during the tests. Moreover, we previously observed a very small and negligible change in inductance under and applied stretch of up to 70% of the inductor’s length in the single sensor LC resonant system featuring a similar inductor (Galli et al., 8 2023). The effect of bending on inductance in the operating frequency range of interest (between 20 MHz and 60 MHz) was an increase in inductance with bending: in particular, with the sharpest bending (radius 30 mm), a percentage increase between 0.5% (at 20 MHz) and 11% (at 60 MHz) was observed (Supplementary Figure S11).

#### 3.1.2 Textile capacitive strain sensors

To calculate the expected capacitance for the sensors, the dielectric constant of the utilized non-conductive spandex was measured: results show a stable value for $\varepsilon_{r}^{\prime }$ of about 1.75 (Supplementary Figure S12), in line with previous literature results (Sankaralingam and Gupta, 2010). Supplementary Table S4 shows the corresponding calculated capacitance values for the chosen length and width ranges.

To select the baseline value for the sensors (and therefore their size), simulations were performed with varying *C*_*1*_ and *C*_*2*_ (circuit schematic in Supplementary Figure S5). With the chosen ranges for length and width, multiple combinations of *C*_*1*_ and *C*_*2*_ were eligible to obtain resonance frequencies distinguishable from each other (Supplementary Table S7). As expected, the further the values of *C*_*1*_ and *C*_*2*_, the larger the distance between the two resonance peaks. A threshold of minimum 1 MHz between the two resonance frequencies was chosen as exclusion criteria as it was observed experimentally that this was enough to clearly distinguish the two *S*_*11*_ peaks, particularly considering that experimentally recorded *S*_*11*_ peaks were inevitably less sharp than (more ideal) simulation data, and therefore a clear separation of the two resonance peaks was needed. All combinations of the two sensors that did not satisfy the condition:

$$f_{res,2}-f_{res,1}>20\;MHz$$

were excluded (shaded in grey in Supplementary Table S6).

The main constraint for the size of the sensors (and therefore the design, length, and width) was in the sensor’s behavior when applied on the garment. Initial tests with sensors integrated in sport leggings showed that for the sensor on the glute a thin and long design was needed to observe a considerable change in capacitance and therefore resonance frequency during the activities, therefore *C*_*2*_ was fabricated with a length of 80 mm and a width of 10 mm. Conversely, the sensor on the kneecap was stretched significantly in most activities (even by simply toning the quad muscle), therefore C_1_ was fabricated with a length of 70 mm and a width of 15 mm. This resulted in baseline capacitance values (measured with LCR meter) of $C_{1,0\;}≅22\;pF$ and $C_{2,0\;}≅17\;pF$ : these values are in line with the expected capacitance from Equation 6 ( $C_{1,0\;}=20.57\;pF$ and $C_{2,0\;}=15.67\;pF$ , Supplementary Table S4).

**FIGURE 2:**
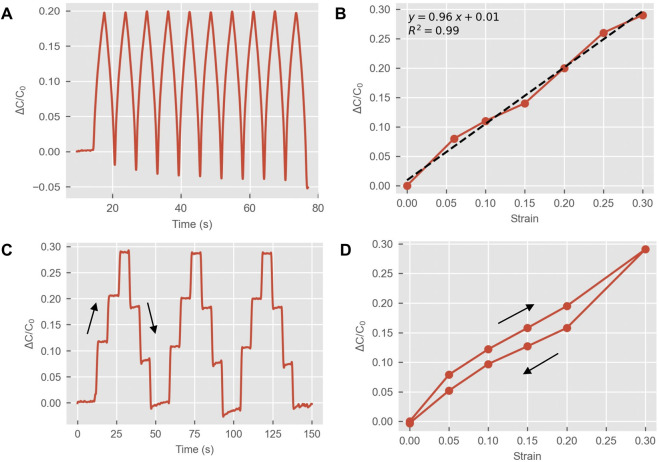
Electromechanical characterization of the textile capacitive sensors. **(A)** Example of cyclical tensile test with 10 cycles of strain application; **(B)** Response to strain up to 30%. Each point is obtained from the average *C* across the 10 cycles of each strain level (as shown in **(A)**); **(C)** Step-hold test with three cycles (10, 20, and 30% strain); **(D)** Results for the step hold test up to 30% strain obtained averaging three cycles (values for 5%, and 10% strain from another step-hold test). The arrows indicate the stretch and release phases in the cycles as in **(C)**.

The electrical response to strain of the textile capacitive sensors is shown in Figure 2 (results from *C*_*1*_). After preconditioning, the sensors showed a stable response, i.e. the same capacitance at maximum applied strain across the 10 cycles (peaks in Figure 2A) with a drift in the baseline value (troughs in Figure 2A) towards lower capacitance due to the relaxation of the fabric after the stretching phase.

A linear response to strain up to 30% was observed, with a gauge factor (sensitivity) around 1 as expected for parallel plate capacitive sensors (Figure 2B). As previously mentioned, values are reported up to 30% strain as this is the range of strain expected when sensors are applied on garments. The step-hold tests (Figure 2C) showed hysteresis–different capacitance or the same level of strain applied in stretch vs. release stages–due to the viscoelastic properties of the fabrics (conductive and non-conductive spandex). Step-hold tests were relevant to observe a behavior like holding a pose in static tests for activity classifications where sensors are stretched and held in a similar stretching state for a few seconds. In the release phases, a higher static drift was also observed (variation in *C* at fixed strain) as compared to the stretching phases.

#### 3.1.3 System response to controlled strain

**FIGURE 3:**
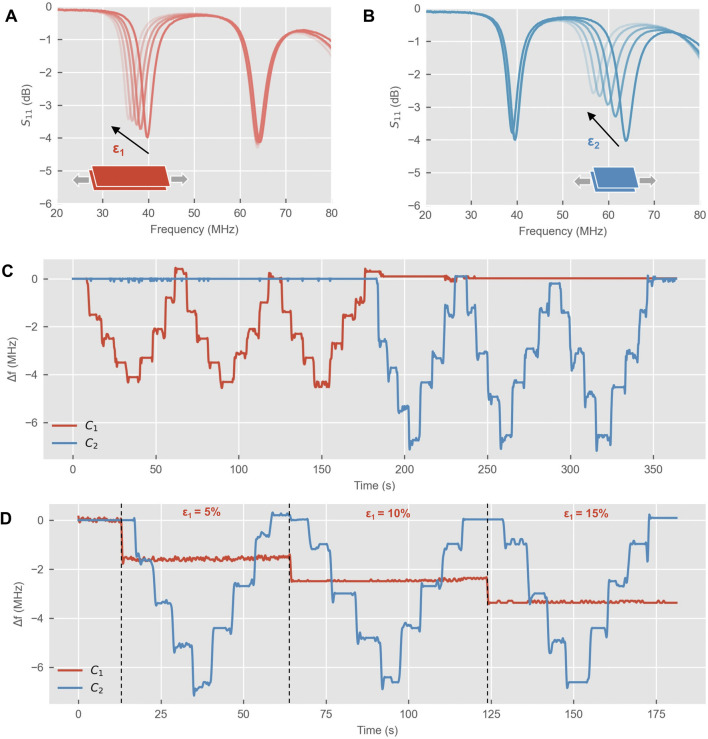
System’s response to controlled strain with two sensors **(A)** Variation of the resonance frequency for sensor 1 (*C*_*1*_) with resonance shifting down as strain increases (ε_1_). The minimum (negative peak) of *S*_*11*_ was selected as resonance frequency for sub-circuit 1. **(B)** Variation of the resonance frequency for sensor 2 (*C*_*2*_) with resonance shifting down with increasing strain (ε_2_). **(C)** Step hold test where one sensor is strained at a time (5%, 10% 15% and 20% strain) and held for about 4 s, then released back to the unstretched state following the same strain values. **(D)** Step hold test with simultaneous stretching of the two sensors (*C*_*1*_ stretched to 5, 10% and 15%, C_2_ stretched and released) with the same pattern as in **(C)**.

The bench tests showed a stable response of the system to strain of each sensor and minimal interference between the two: stretching only one sensor resulted in a shift in resonance of the corresponding sub-circuit, but not in the other sub-circuit (Figures 3A,B). Step-hold tests with sequential stretching of the two sensors also showed a repeatable response across three cycles (Figure 3C). Additionally, stretching both sensors confirmed these findings as shown by stretching and holding *C*_*1*_ and simultaneously stretching *C*_*2*_ to various levels of strain. Figure 3D shows the measured resonance frequencies as *C*_*1*_ is stretched and held (5, 10% and 20%) and *C*_*2*_ is progressively stretched: as f_res1_ remains stable with stable strain on sensor *C*_*1*_, *f*_*res2*_ varies according to the strain applied on sensor *C*_*2*_. The difference in resonance between the stretch and release phases reflected the behavior of the capacitive sensors observed in the electromechanical tests: in the release phase, a lower capacitance was recorded with respect to the stretching phase for the same level of strain (Figure 2C), which resulted in a higher resonance frequency of the sub-circuit visible for both sensors (Figure 3C). Of note, having designed the system in such way that *f*_*res2*_ *- f*_*res2*_ *> 20 MHz*, the two resonance peaks were far enough in the frequency spectrum that even if in the release phase both resonance frequencies were higher than the stretch phase, the two peaks would still not overlap.

The variation in resonance frequency for *C*_*2*_ was larger than that for *C*_*1*_ for the same strain as expected from Equation 1 ( $f\propto 1/\sqrt{C}$ ) and was also confirmed by the simulations. Experimental results showed slightly higher *Δf* for both sensors as compared to the simulations (Supplementary Figure S12), especially for sensor 1 (Supplementary Table S9). This discrepancy may be due to small experimental effects not being fully accounted for in the simulations, such as movement of the sensors when stretching them manually with the 3D printed fixtures and readjustment of the fabric layers. However, the overall behavior of the system was qualitatively consistent with the simulations.

### 3.2 Activity classification with sensorised garment

**FIGURE 4:**
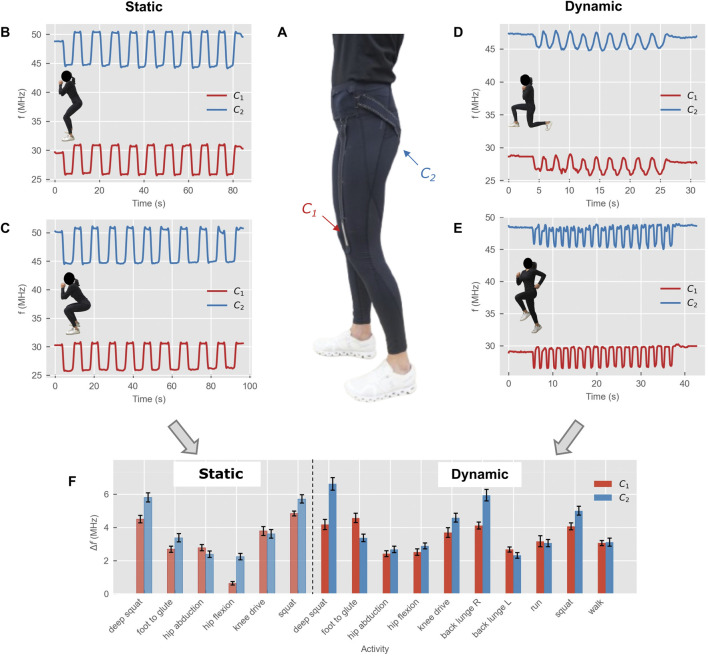
Human movement monitoring with sensorised sport leggings. **(A)** Front view of the sensorised sport leggings showing the first sensor on the kneecap. **(B–E)** Recorded resonance frequency shifts for some of the activities: static (**(B)** Squat, **(C)** Deep squat), and dynamic (**(D)** Lunge, **(E)** Run); **(F)** Resonance frequency shifts for all activities (mean ± SD across the 10 cycles, and across steps for running and walking). Shaded areas (leftmost bars) indicate the static tests.

To demonstrate the capability of the system to track human movement and classify different poses and activities, sensorised sport leggings with two sensors on the left leg (*C*_*1*_ on the kneecap, *C*_*2*_ on the glute) were used (Supplementary Figure S7). Several activities were performed and the response of both sensors in terms of resonance frequency of the corresponding sub-circuit was recorded. The goal was to observe how the response of each sub-circuit changed with different degrees of knee and hip flexion (and hence different stretching of each sensor). Figure 4 shows part of the recorded activities performed in static tests (pose held for 4 s) and dynamic tests (continuous repetitions). The complete results for all tests are shown in Supplementary Figure S14 (static tests) and Supplementary Figure S15 (dynamic tests). From the absolute values of *f*_*res1*_ and *f*_*res2*_ can be observed that the baseline resonance frequencies of standing phases could vary among tests: this is to be expected as any repositioning or adjustment of the sensor would naturally slightly change its capacitance and therefore the resonance frequency of the corresponding sub-circuit. However, since the parameter of interest is the resonance frequency shift with strain, deviation in the baseline values were not relevant.

For both sensors, higher resonance frequency corresponds to the standing phases (unstretched sensors, lower capacitance) and as the sensors are stretched the capacitance increase drives down the resonance. Each sensor (*C*_*1*_ and *C*_*2*_) was stretched to a different extent based on the activity, and the corresponding change in resonance frequency of the sensor’s sub-circuit could be used to differentiate the activities.

For all tests, the standing phases showed higher variability among cycles as compared to the activity phases, possibly due to the relaxation of the fabric of the textile sensors in the release phase after the stretching, similarly to what was observed in the electromechanical response of the capacitive sensors (Figure 2). Standing and activity phases were labeled using threshold calculated using Equation 10 and Equation 11, respectively. Despite most phases being labeled correctly (e.g., deep squat, Supplementary Figure S16A), for some tests part of the automatically detected labels was incorrect, meaning that data points corresponding to either “standing” or “activity” were labeled as “transition”. To reduce manual relabeling as much as possible, two different approaches were adopted for incorrectly labeled datapoints: i) if the correct number of cycles could still be detected, but some datapoints in one or more cycles were incorrectly labeled, the labels were left as were (e.g., hip abduction, Supplementary Figure S16B); ii) if all datapoints in a “standing” or “activity” cycles were incorrectly labeled as “transition” and therefore the cycle was not correctly detected, the labels were manually adjusted (e.g., run, Supplementary Figure S16C). The latter case only occurred for the following tests: hip flexion (static and dynamic), walking, running.

The ranges of resonance frequency shifts for both sensors across all activities are shown in Figure 4F, with mean values and standard deviation across the cycles (10 cycles for all activities, 20 steps for running and walking). Variability of the response across cycles was slightly more pronounced in dynamic tests, with maximum observed standard deviation of 0.33 MHz and 0.38 MHz for static deep squat and dynamic deep squat, respectively. This variation could be attributed to the hysteresis of the sensors and viscoelasticity of the textile materials as observed in the different response of the system to stretch and release phases in the bench tests (Figures 3C,D). The best approach to limit this phenomenon is to ensure that both sensors are placed on the garment in a taut configuration when no stretch is applied, that is, in the standing phases. In this way, slacking of the sensors in the release phase after stretch is minimized, therefore limiting the artificial change in capacitance due to uncontrolled distance between the electrodes.

For the same activity, dynamic tests generally showed slightly higher resonance frequency shift as compared to the static tests (Figure 4F): this could be attributed to the lower level of control of the movement and more overall movement of the circuit components (connection lines and sensors) during the activity.

Among the explored features, a total of 11 features were retained (five per sensor plus the correlation between the two sensors): mean, standard, deviation, RMS, range, entropy, and correlation between the two sensors.

As for the classification task, a clear distinction among every activity was observed in the resonance shifts (Figure 4F), and correspondingly all machine learning models showed high performance (Table 1). The highest accuracy and F1-scores were 0.98 and 0.98 respectively for static activities, and 0.96 and 0.96 respectively for dynamic activities. Such results are in line with previous works in the literature for human activity recognition/classification with wearable textile sensors: upper body activities were classified with 0.97 accuracy for leave-session-out and 0.86 accuracy for user independent recognition with a blazer equipped with capacitive sensors (Bello et al., 2021), and 0.90 F1-score was reported using a shirt equipped with 16 sensors on 21 participants (Zhou et al., 2023). For lower body activity classification, an accuracy of 0.96 for the distinction of four phases of gait was obtained with a sock including four capacitive pressure sensors (Vu and Kim, 2020), and 0.90 with a single strain sensor on tight shorts for distinguishing walking, jumping, running, and sprinting (Vu and Kim, 2018). Another study on gait phases (only three phases) using ten textile pressure sensor on tight leggings reported an accuracy of 0.93 (Milovic et al., 2022). A complete overview of the aforementioned studies and the corresponding classification performance is available in Supplementary Table S1 for comparison.

**TABLE 1:** Classification results for static and dynamic tests.

Model	Static		Dynamic	
​	Accuracy	F1-score	Accuracy	F1-score
K-nearest neighbors	0.98	0.98	0.89	0.89
Logistic regression	0.96	0.94	0.91	0.91
Support vector machine	0.99	0.98	0.96	0.96
Decision trees	0.98	0.98	0.83	0.81
Random forest	0.98	0.98	0.96	0.96
Extreme gradient boosting	0.96	0.94	0.93	0.92

Classification performance was better for static tests compared to dynamic tests as the signals were overall cleaner for the static tests, and for dynamic tests some standing and activity phases were short (containing only a few samples). As some classes (i.e., activities) had fewer samples than others (due to the threshold-based labeling, more noisy data resulted in more “transition” phases than other datasets), F1- score is also reported as a measure of performance. This metric is particularly appropriate to account for imbalance in the dataset as it considers both precision (higher precision: low false positives) and recall (high recall: low false negatives).

Generally, high accuracy is expected with a single participant dataset and given that all cycles of the same activities were repeated in the same way and during a single session. However, the observed performance confirmed the capability of the system to track movement in a consistent manner across multiple cycles of the same activity, which is the starting point to prove the suitability of the sensorised garments for the activity classification task.

To assess the generalizability of results to multiple independent sessions with a single participant, the same protocol was performed on three different days. Although his is not equivalent to testing on multiple participants, doffing and donning the prototype on different days significantly increases the variability of the dataset: sensors are inevitably worn in a different initial stretch state as the leggings fit differently. Activities are also performed in a slightly different way as movements are not constrained (free-body exercises). As a result, both baseline shifts and variations in signal amplitude across sessions are expected.

Such inter-session variability introduces a distribution shift between training and testing data, which is known to negatively affect classification performance as also noted in other works assessing the effect of sensor positioning and displacement among multiple sessions or participants (Banos et al., 2014; Khaked et al., 2025). Consequently, a drop in classification performance is expected, especially for models that are less capable of capturing nonlinear decision boundaries or that rely on distance-based similarity in feature space. As shown in Table 2, the largest degradation in performance was observed for Logistic Regression, reflecting its reliance on linear separability. This was followed by k-Nearest Neighbors and Support Vector Classification. In contrast, tree-based and ensemble (boosting) models showed higher robustness to inter-session variability, with the best performance obtained with Random Forest.

**TABLE 2:** Classification results for dynamic tests on all three sessions.

Model	Dynamic	
​	Accuracy	F1-score
K-nearest neighbors	0.75	0.74
Logistic regression	0.55	0.55
Support vector machine	0.80	0.80
Decision trees	0.81	0.82
Random forest	0.86	0.87
Extreme gradient boosting	0.85	0.84
Light gradient boosting	0.85	0.85
Categorical boosting	0.85	0.84

To the best of our knowledge, there are no reports of classification performance with donning and doffing textile-based sensing systems and recording multiple sessions on different days. However, the obtained classification results are in line with previous literature on human activity classification with wearable textile sensors, as discussed above and shown in Supplementary Table S1.

When comparing to activity classification with IMU sensors, some reported nearly perfect (0.99 accurate) classification of running exercises using four sensors (Müller et al., 2024), and generally high accuracy (>0.90) can be attained with multiple IMU sensors as summarized by a study presenting an adaptive tree-based algorithm (Nematallah and Rajan, 2024). Others reported lower performance close to what we have obtained in the multi-session analysis. A study with five IMU sensors reported participant specific F1-score in activity recognition (lifting and lowering a box, standing, walking) of 0.90 across 12 participants (Pesenti et al., 2023). Similarly, using only one or two IMU sensors (on upper and lower leg, single side) yielded F1-scores of 0.89 and 0.91 respectively in classifying phases of gait (Marcos Mazon et al., 2022). Of note, studies with IMU sensors have a larger dataset with multiple participants as the main focus of the study is the development of efficient algorithms for human activity recognition and the data is acquired with commercial IMU sensors. In the case of textile based sensing system, generalizing to multiple participants is more challenging as it includes finding the appropriate size and fitting of the prototype (here sensorised leggings) and accounting for differences in the signals due to prototype fitting and sensor positioning.

Overall, activities could be successfully classified both in static and dynamic conditions with relatively simple standard machine learning models and with a limited dataset, showing initial promise for the use of the wearable device in activity detection tasks.

This study serves as proof of concept for the multi-sensor wireless sensing system and was performed with a single participant. The validation reflects within-participant, within-session generalization and may overestimate performance in cross-participant or real-world deployment scenarios. Therefore, future developments include performing the same tests on a larger number of participants to evaluate the real generalizability of the models: this would allow generalization of the results considering the variability between participants, for example due to anatomical differences and different fit of leggings.

Moreover, other activities and poses could be explored where the stretching of the two sensors is less evident and therefore a higher noise in the data could be expected. These preliminary results show that the system is in principle suited for activity classification with simple machine learning models. Real-world deployment would require robustness to inter-participant variability, sensor placement differences, and more subtle movement. In this perspective, the expansion to multiple participants would also allow the use of more powerful machine learning and deep learning approaches that typically require a bigger and more variable dataset.

As for the sensorised garment’s robustness to environmental conditions, we have previously conducted tests on the effect of different temperature and humidity that showed an overall consistent response of the wireless textile circuit to such conditions, with variations in the frequency shift at the same strain of less than 0.5 MHz up to 10 washes and almost no change with temperatures between 15  °C and 40  °C and humidity between 20% and 75% (Galli et al., 2023). Preliminary tests on the response of the (single sensor) wireless textile system to the application of artificial sweat also showed promising results, with a measurable response (frequency shift) both in damp and dried out conditions (Galli et al., 2024).

A technical improvement of the system would be the use of a portable reader instead of benchtop VNA, allowing the use of the textile sensing device outside of the laboratory. There exist portable and inexpensive versions of VNAs, but a key limitation is in the sampling frequency: none of the currently available portable 5NA can reach a sampling rate above a few Hertz. We have attempted recording static activities with a portable 5NA (VNA6000, NanoRFE, China) with a dedicated application programming interface (API) to increase the default sampling frequency but could not reach $f_{sampling}>3-5\;Hz$ , even reducing the number of sweep points and the range for the frequency sweep to the minimum (just slightly below $f_{res1}$ and above $f_{res2}$ ). Such low sampling frequency severely limits the application of the system, as only slow static activities could be recorded and even then, the data quality would be insufficient to distinguish small resonance frequency shifts.

Such limitations could be addressed by optimization of the reader hardware (available portable 5NAs) to increase sampling rate. Alternatively, modification and optimization of our previously developed electronics module for real-time resonance frequency tracking of a single sensor (“fReader”, Galli et al., 2023) could be an interesting approach to have a lightweight reader communicating with a smartphone for a truly wearable solution.

Lastly, the multi-sensors textile system could be further expanded to include yet more sensors, for fine movement capturing or more complex activities involving, for example, movement of joints in 3D (e.g., hip movement in the frontal or lateral planes). To include more sensors, a careful design of the textile inductor and capacitive sensor values (e.g., with LT spice simulations) would be crucial to ensure minimal interference among sensors while still having distinguishable resonance peaks. Moreover, including the number of resonance peaks (one per sensor) would require extending the frequency range for each sweep to have a clear separation among them (e.g., above 20 MHz as mentioned above). Maintaining the same resolution in frequency (i.e., the number of points per sweep) and at the same time a high sampling frequency could become challenging even using a high-end benchtop VNA.

## 4 Conclusion

In this work, we have presented a proof-of-concept all-textile system with embedded sensing capabilities able to track body movements and transmit this information wirelessly to an external reader. The data transmission and powering of the system are based on inductive coupling with an external reader and the textile sensing circuit on the garment is fully passive. The sensing modality is based on multiple LC resonant circuits with capacitive strain sensors connected to a single textile inductor: the stretching of the sensor during movement modulates the resonance frequencies of each sub-circuit (one per sensor) which are tracked by an external reader (VNA).

The system is an expansion of our previous development featuring one capacitive strain sensor: by including multiple sensors in various locations on the garment, we demonstrated the feasibility of classifying different activities beyond the simpler single joint tracking with a single participant and multiple independent sessions. Such development opens the possibility to use the system in different fields of application where continuous and unobtrusive movement monitoring is essential, from rehabilitation settings to sports.

In this work, two sensors were used and both sensors were put on the same leg to track both the knee and hip joints. Further expanding to more sensors would allow to increase resolution around the joints (by putting all sensors on a single leg, for example two around the knee and two around the hip) or include both legs to observe any variability between left and right leg in symmetric exercises and further expand the activity classification to other movements. Of note, contrary to most systems in the literature with multiple sensors that use wired connections to a rigid electronic board, the wireless setup is advantageous to remove such rigid components but makes the use of multiple sensors more challenging, as interference among sensors becomes problematic with more sensors.

The components of the passive textile sensing circuit can be easily manufactured with existing commercial materials and simple textile machinery (sewing, embroidery). As previously demonstrated with the single sensor system, the sensors and textile inductor can be placed at various locations on garments with little adaptation and therefore be used for movement tracking of different body parts depending on the application at hand.

Common activities such as walking, running, or typical exercises (squat, hip abduction or flexion, knee drive, *etc.*) could be successfully classified in a single participant setting, showing the feasibility of the system as a flexible, all-textile wearable device for movement monitoring and activity recognition. Future work is required to assess the robustness across multiple users and sensor placements.

Overall, this study established the feasibility of a passive, all-textile wearable system to monitor movement wirelessly and continuously, without physical constraints of the laboratory space or hindrance of rigid components of the alternative available technologies and therefore suitable for diverse application in sports or clinical fields.

## Data Availability

The raw data supporting the conclusions of this article will be made available by the authors, without undue reservation.
