# Supplementary material for: Fully textile passive wireless sensing for human movement monitoring with multiple sensors
Source: Front Bioeng Biotechnol. 2026 Feb 25;14:1724364. doi: 10.3389/fbioe.2026.1724364 (PMC12975977; doi:10.3389/fbioe.2026.1724364)
Supplement: Supplementary file 1 [file DataSheet1.pdf]

Supplementary Material

Supplementary Figures

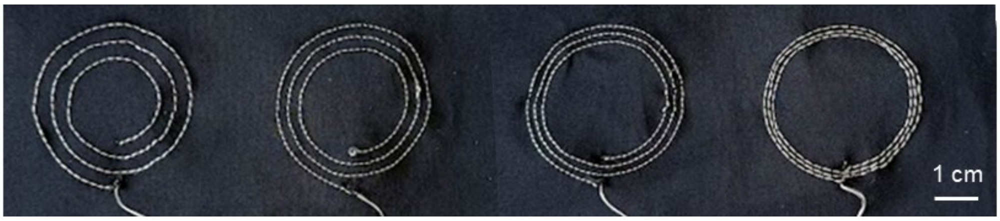

Supplementary Figure 1. Textile inductors with outer diameter $d_{out} = 40\ mm$ and varying gap (from left to right 4 mm, 3 mm, 2 mm, 1 mm).

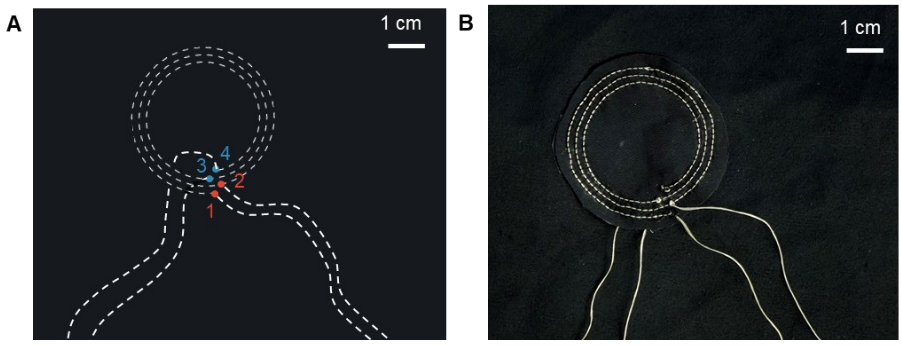

Supplementary Figure 2. A: Illustration of the tapping points on a three-turn inductor (connections 3 and 4 running on the back side of the inductor); B: Photograph of inductor with gap 2 mm and tapped connections.

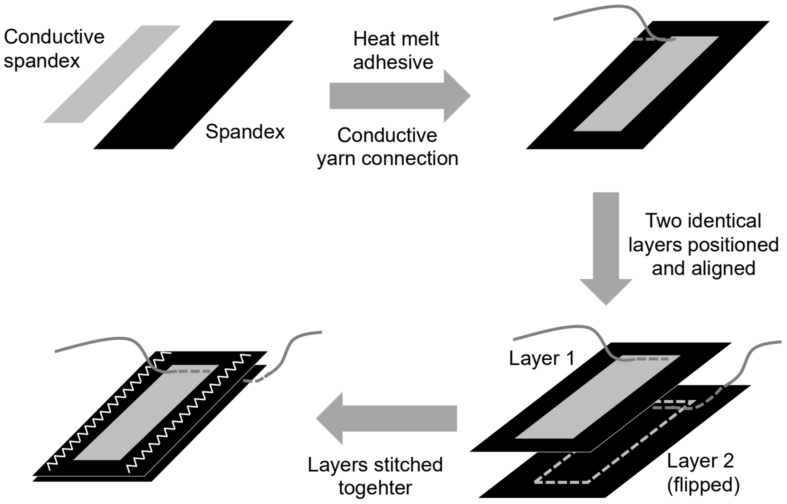

Supplementary Figure 3. Fabrication of the textile capacitive strain sensors: the conductive spandex electrodes were attached to a non-conductive spandex fabric with a heat melt adhesive web and conductive yarn was stitched to the electrode to make electrical connection. Two identical layers (the two parallel plates of the capacitors) were overlapped aligning the electrodes and with non-conductive spandex sides facing each other. Lastly, the two layers were stitched together with zigzag stitching to allow stretching of the sensor along its length.

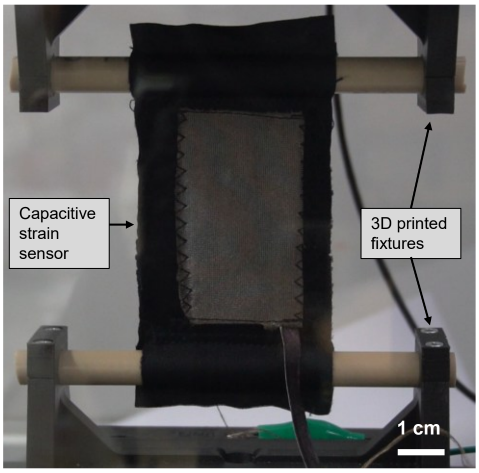

Supplementary Figure 4. Capacitive strain sensor mounted on the UTM machine using custom 3D printed fixtures: the sensors were secured to the fixtures with polyether ether ketone (PEEK) rods inserted in stitched loops in the fabric. Each electrode was connected to a terminal of the inductance-capacitance-resistance (LCR) meter with a short connection made of the same conductive yarn (Liberator 40-Ag) used for the whole system (textile inductor and connections).

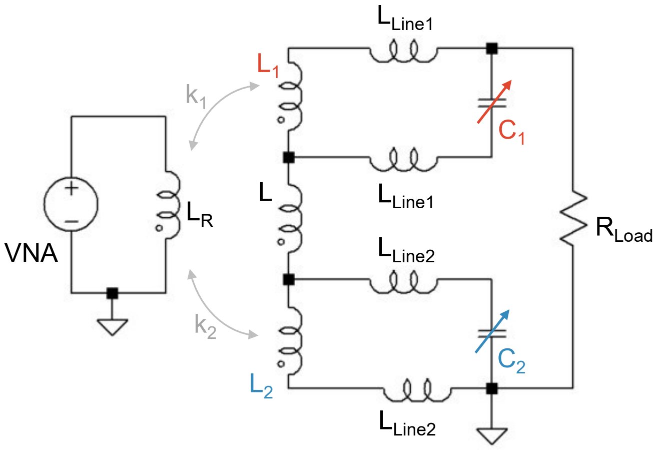

Supplementary Figure 5. Circuit schematics for the *LT spice* simulations.

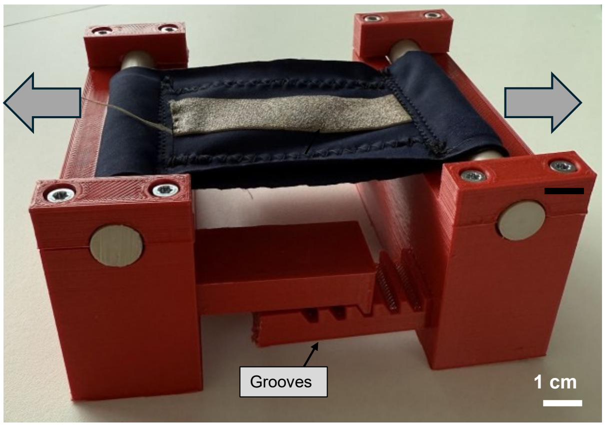

Supplementary Figure 6. Capacitive strain sensor mounted on a 3D printed fixture for bench tests with simultaneous stretching of multiple sensors: the sensor was stretched by pulling the two sides away (direction indicated by the grey arrows) and using the custom-made grooves to fix them in place.

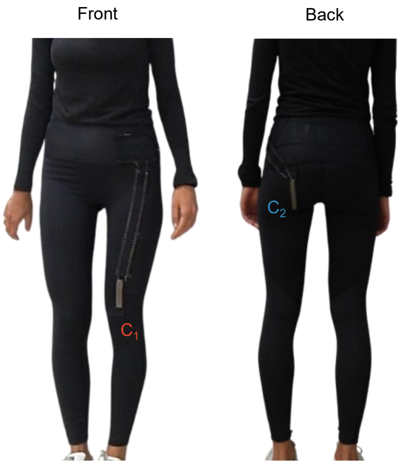

Supplementary Figure 7. Sensorised sport leggings used for the activity classification tests with two sensors: C_1 above the kneecap and C_2 on the glute.

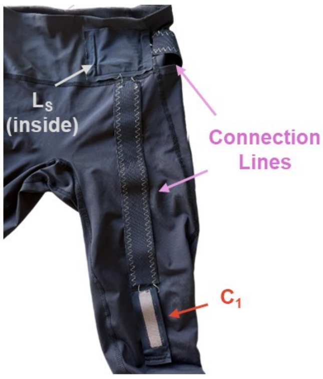

Supplementary Figure 8. Close-up view of the electrical connection lines to between the inductor inside the pocket (L_S) and the sensor on the kneecap (C_1). The connection lines on the waistband running to the back connect L_S to C_2 on the back of the leggings (as visible in **Supplementary Figure 7**).

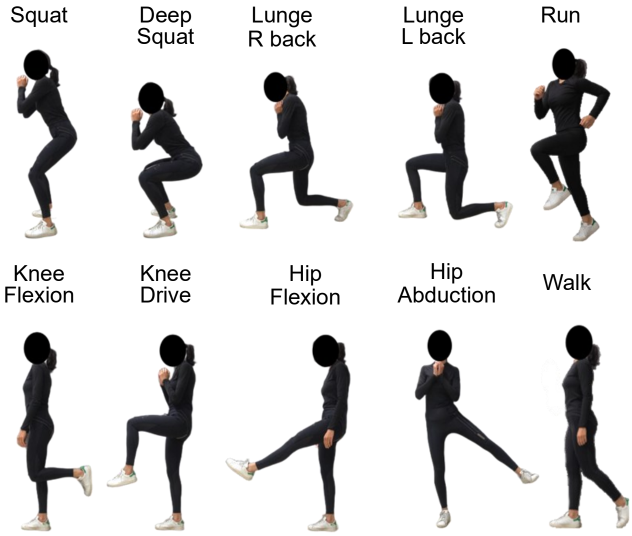

Supplementary Figure 9. Illustration of all the activities performed in the tests with sensorised sport leggings.

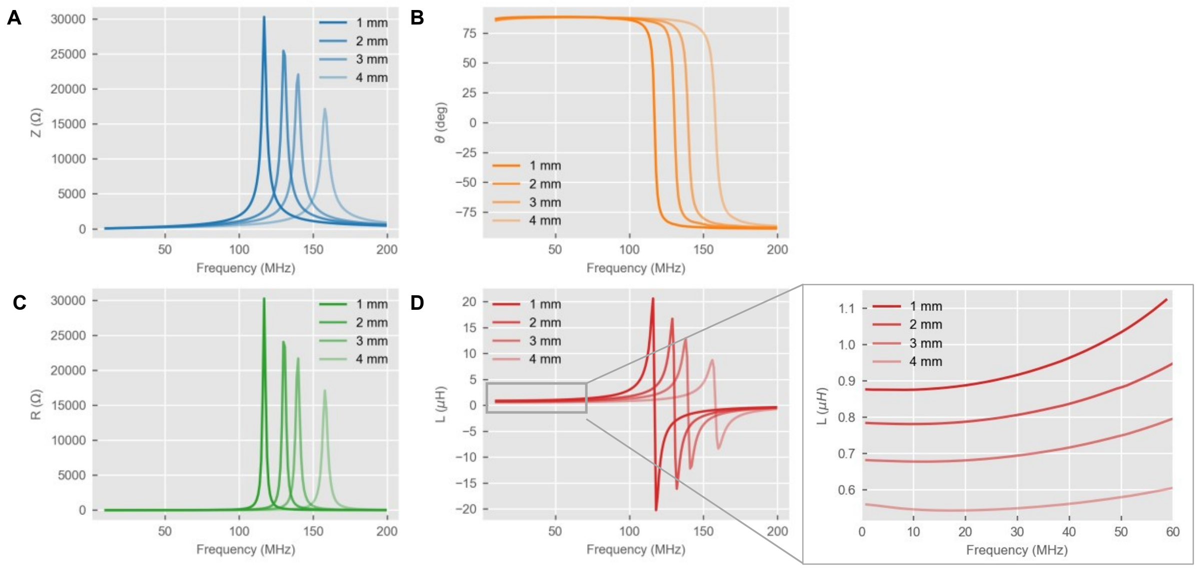

Supplementary Figure 10. Impedance profile of the textile inductors with varying gap (s = 1 mm to s = 4 mm). **A**: Impedance magnitude; **B**: Impedance phase; **C**: Equivalent series resistance; **D**: Inductance.

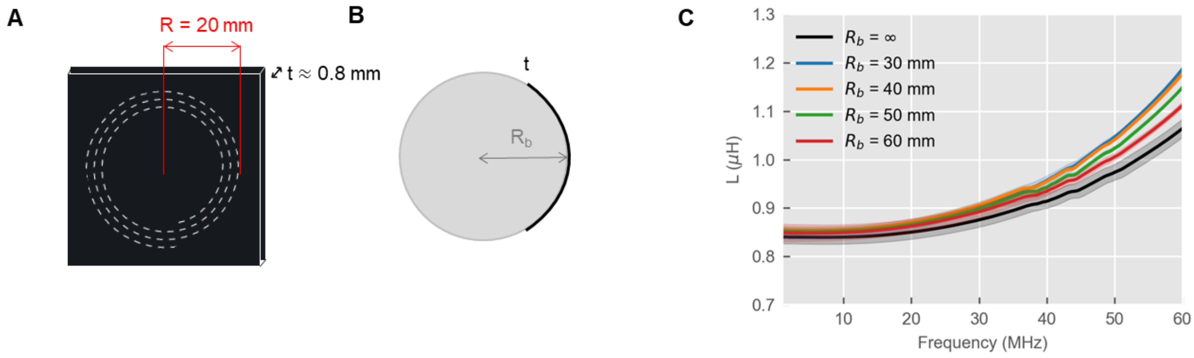

Supplementary Figure 11. Effect of bending on the textile inductor. **A**: schematic illustration of the chosen inductor (s = 2 mm); **B**: illustration of the bending test where the inductor is placed on a cylindrical surface of different bending radius R_b; **C**: inductance in the frequency range of interest for different bending radii (mean ± SD, 3 tests). For the sharpest bending radius R_b=30 mm, L_{max}(f=60MHz)=1.19 μH, which is 11% higher than baseline at $R_b=\infty$ (L_{max}(f=60MHz)=1.07 μH).

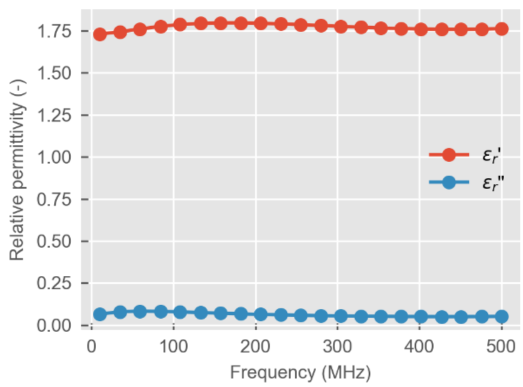

Supplementary Figure 12. Dielectric properties of non-conductive spandex for the textile capacitive sensors (dielectric layer) displaying the real and imaginary parts of the complex relative permittivity. The real part ε'_r (referred to as dielectric constant) is the most relevant for capacitors and describes the ability of the material to become polarized by an electric field and store electrical energy. The imaginary part ε''_r is related to energy dissipation through heat (dielectric losses)

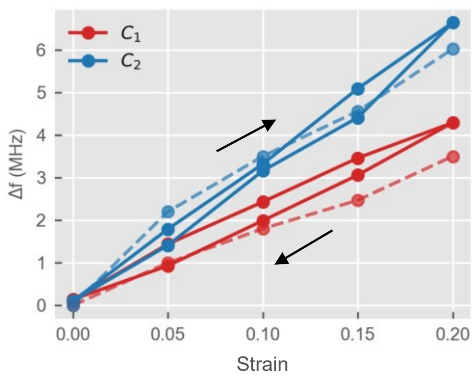

Supplementary Figure 13. System response to controlled strain with variation in resonance frequency for each sensor at 5, 10, 15 and 20% strain (average of three cycles of step hold tests). Dashed lines indicate the simulation results. For the measurement results (solid lines) the stretch and release phases are shown according to the results shown in **Figure 3C**. Simulation values do not account for the difference between stretch and release phases, therefore the responses for stretch and release phases are overlapped.

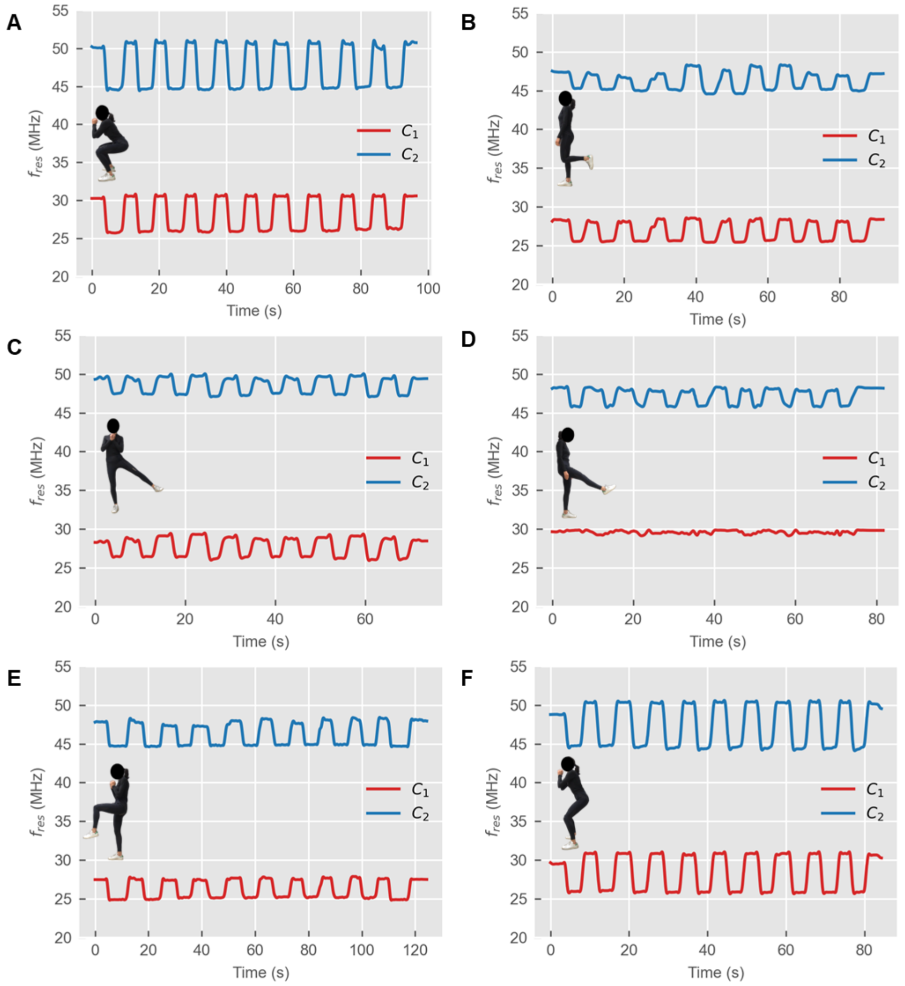

Supplementary Figure 14. Results from the *static* tests with sensorised sport leggings. The signals represent the resonance frequencies of sub-circuit 1 (sub-inductor 1 and sensor 1 (C_1) on the kneecap and sub-circuit 2 (sub-inductor 2 and sensor 2 (C_2) on the glute. **A**: deep squat; **B**: foot to glute; **C**: hip abduction; **D**: hip flexion; **E**: knee drive; **F**: squat.

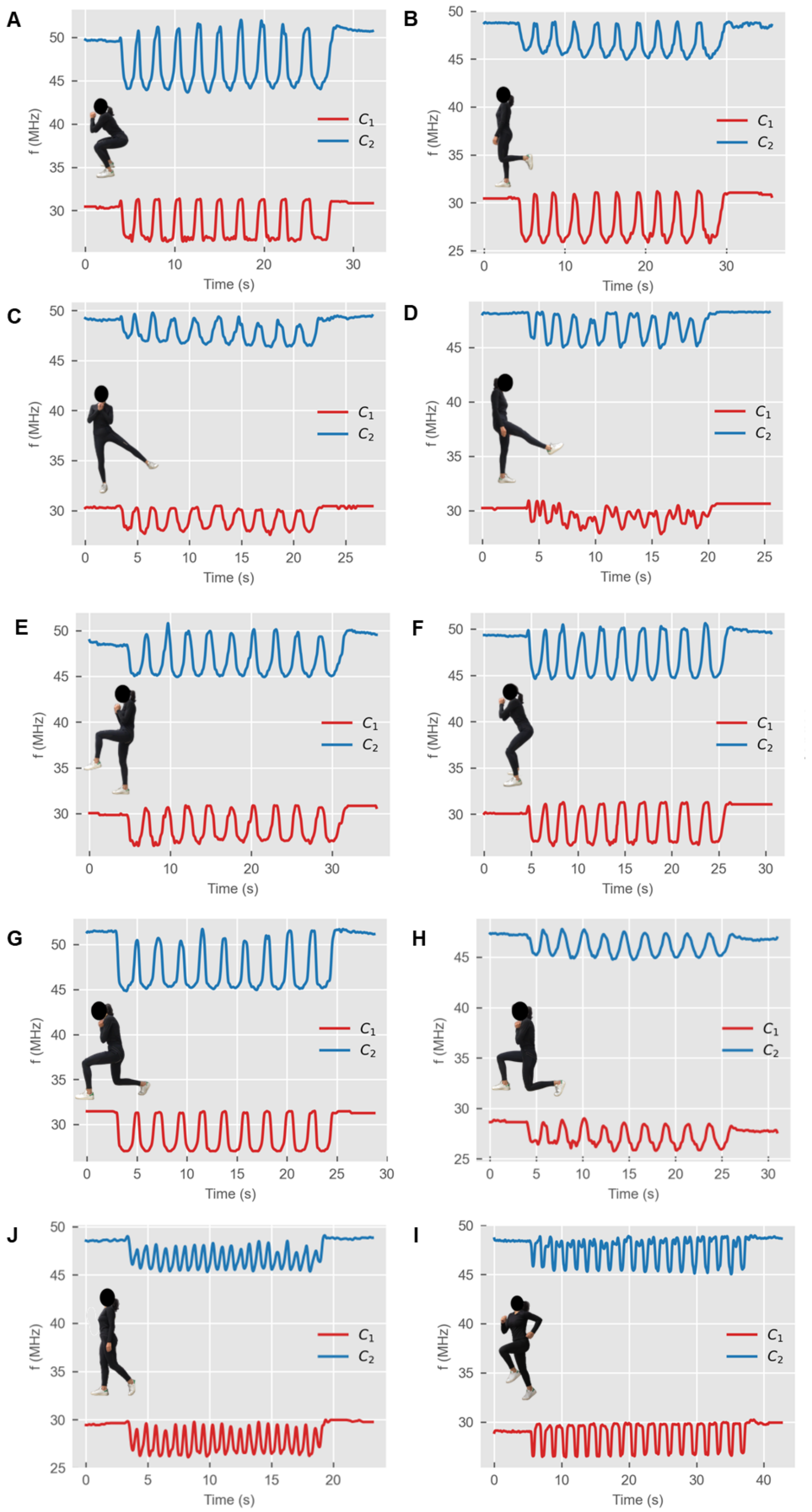

Supplementary Figure 15. Results from the *dynamic* tests with sensorised sport leggings. **A**: deep squat; **B**: foot to glute; **C**: hip abduction; **D**: hip flexion; **E**: knee drive; **F**: squat; **G:** lunge with right leg back**; H:** lunge with left leg back **I:** walk **J:** run**.**

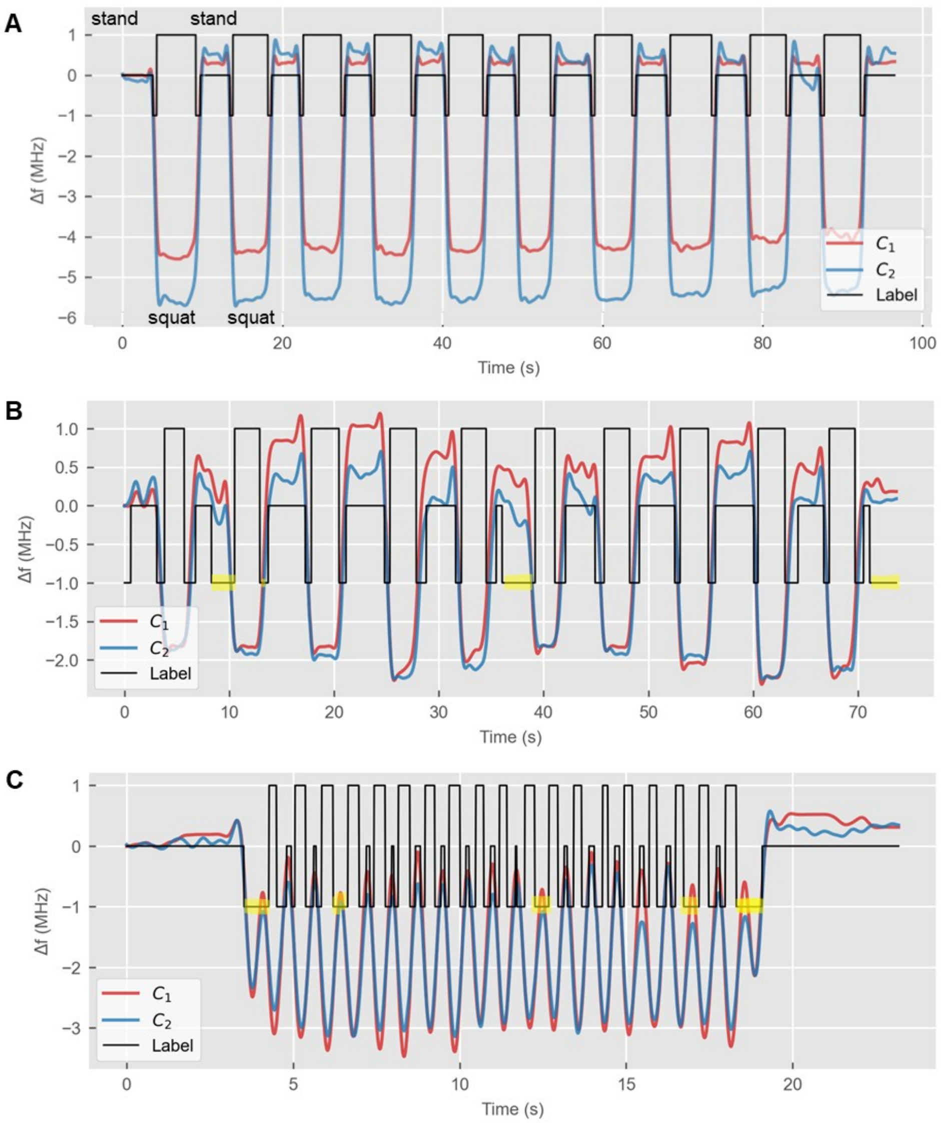

Supplementary Figure 16. Example of the labelling approach for tor different tests: label 0 was used for the "standing" phases, label 1 was used for the "activity" phases and label -1 for the "transition" phases in between. The normalized resonance frequency ($\Delta f = f - f_0$) is reported for ease of visualization, as f_1 and f_2 follow in different ranges. **A**: Deep squat (static test): all labels correct, 10 cycles are detected; **B**: hip abduction (static test) incorrectly labelled phases are highlighted in yellow: for these sections of the test, neither sensor was labelled correctly as the corresponding resonance frequency value fell outside of the threshold (specifically the standing phase that should be labelled 0 is labelled as transition -1). However, 10 cycles are correctly identified; **C:** Run (dynamic test): incorrectly labelled phases highlighted in yellow, in this case some cycles were missed and therefore labels have been manually adjusted.

Supplementary Tables

Supplementary Table 1: Overview of textile-based systems for activity recognition or movement monitoring in the literature. Studies reporting joint angle position and/or angles are highlighted in grey, all other studies are based on activity recognition/classification.

In the "Prediction Model" column, if several models were used only the best performing one is reported with its corresponding accuracy ("Performance" column).

Sensing modality	Study	Form Factor	Sensor	Readout method	N. S.	N. P.	Application	Prediction Model	Performance
Capacitive	**This work**	**Tight fitting leggings**	**Strain sensor, conductive stretchable textile**	**Wireless, inductive coupling**	**2**	**1**	**Lower body 10 poses and exercises**	**SVM and RF**	**F1-score 98% for static, 96% for dynamic**
	MoCaPaci (Bello et al., 2021)	Loose fitting blazer	Commercial textile cables as antennas coupled to the body (Theremin device)	Two Open Theremin boards	4	14	Upper body 20 poses (4 random repetitions per pose)	CNN 2D	Accuracy 97.18% for LSO, 86.25% for LPO
	MoCaPose (Zhou et al., 2023)	Loose fitting shirt	Conductive textile patches coupled to the body	Custom electronic board on the jacket	16	21	Upper body 10 poses	Unsupervised learning and autoencoder	F1-score 79% for static poses, 90% on short time windows
	SeamPose (Yu et al., 2024)	Loose fitting shirt	Conductive yarn in seams coupled to the body	Custom electronic board on shirt	8	12	Upper body 7 poses + dancing and random poses	LSTM	MPJPE 6.0 cm.
	(Cheng et al., 2010)	Tight fitting neck collar	Conductive textile patches coupled to the body	Custom electronic board (unclear if on the collar or not)	3	3	Respiration mode (deep, shallow); swallow, chew, nod, look to the side	LDA	Accuracy 69% while walking, 77% while sitting

Continued

Sensing modality	Study	Form Factor	Sensor	Readout method	N. S.	N. P.	Application	Prediction Model	Performance
Capacitive	(Jin et al., 2020)	Tight fitting shirt	Two stacked parallel plate	Custom electronic board on the shirt	8	1	7 different movement of the shoulder	Catboost	RMSE<4.5 degrees (shoulder)
	(Vu and Kim, 2020)	Sock	Parallel plate pressure sensors	External custom electronic board (wires)	4	1	4 phases of gait	ANFIS	Accuracy 96%
	(Galli et al., 2023)	Tight fitting knee sleeve	Conductive textile patches coupled to the body	Tethered connection to LCR meter	4	1	Knee flexion/extensi on, squat	RF	RMSE=4.15 deg (knee sagittal angle)
	VersaPants (Kasap et al., 2025)	Loose fitting pants	Conductive textile patches coupled to the body	Custom electronic board on the waistband	14	11	Lower body 16 poses and exercises	CNN 2D + transformer	MPJPE=11.96 cm, MPJAE=12.3 deg (hip, knee, ankle)
Resistive	(Vu and Kim, 2018)	Tight fitting shorts	Strain sensor	Custom electronic board	1	1	Walking, jumping, running, and sprinting	RF	Accuracy 90%
	(Mokhlespour Esfahani, Nussbaum, 2019)	Tight fitting T-shirt	Screen-printed strain sensors; commercial smart sock	Custom external electronic board (connected with wires)	11	11	11 classes, walking and running at different speeds + sit, stand and lie down	KNN	Accuracy 96% (with shirt only)
	(Xu et al., 2022)	Tight fitting arm sleeve	Matrix of pressure sensors (knitted)	Custom electronic board attached to the sleeve	200	14	18 activities (10 repetitions)	SVM, CNN	Accuracy 82%, 75% in LPO

Continued

Sensing modality	Study	Form Factor	Sensor	Readout method	N. S.	N. P.	Application	Prediction Model	Performance
Resistive	(Milovic et al., 2022)	Tight fitting leggings	Pressure sensors (conductive fabric and carbon coated polymer)	Custom electronic board on leggings	10	3	3 phases of gait	Random Forest	Precision 91.2%
	(Ma et al., 2024)	Tight fitting shirt	Pressure sensor made of woven custom-made fibers	Custom electronic board	16	1	Sitting posture classification, 8 different postures	CNN + Vision Transformer	Accuracy 98% accuracy
	(Zhang et al., 2023)	Tight knee sleeve	Pressure sensors	Custom electronic board	14 + 2 IMU	6	Squat (various types), hamstring curls, leg raises	LSTM	RMSE=7.21 degrees across all lower body joints
Inductive	(Tavassolian et al., 2020)	Tight fitting shorts	Elastic fiber core with copper twisted around	Custom electronic board	4	12	Running at constant speed	RF	RMSE=2 degrees for in 3D hip angle

Abbreviations:

N. S. = Number of Sensors
N. P. = Number of Participants

ANFIS = Adaptive Neuro-Fuzzy Network;
CNN = Convolutional Neural Network;
KNN = *k*-Nearest Neighbors;
LDA = Linear Discriminant Analysis;
LSTM = Long Short-Term Memory;
RF = Random Forest;
SVM = Support Vector Machine

LPO = Leave-Participant-Out;
LSO = Leave-Session-Out;
MPJAE = Mean Per-Joint Angle Error;
MPJPE = Mean Per-Joint Position Error

Supplementary Table 2. Inductance values calculated from **Equation 3** with the chosen outer diameter, gaps and number of turns compared to measurements from the impedance analyzer. The measured value was taken as the average inductance in the range of frequency below half of the self-resonance frequency (range of operation).

Calculated					Measured
d_{out} (mm)	gap (mm)	d_{in} (mm)	N	L (µH)	L_{mean} (µH)
40	1	35	3	0.77	0.94
40	2	30	3	0.57	0.84
40	3	24	3	0.44	0.73
40	4	20	3	0.34	0.58

Supplementary Table 3. Impedance characteristics of the sub-inductors for the textile inductor L_s with gap *s=2 mm*. L_1 is the sub-inductor made with the first turn from out to in (points 1 to 2 in **Supplementary Figure 1**), L_2 between points 2 to 3, L_3 between points 3 and 4.

Sub-inductor	L (µH)	C_{par} (pF)	f_{self} (MHz)
L_1	0.298	1.43	243.93
L_2	0.250	2.00	208.37
L_3	0.211	1.70	236.35

Supplementary Table 4. Capacitance values calculated from **Equation 6** with the chosen ranges for width and length. The value for permittivity was calculated based on the average ε_r value measured for non-conductive spandex $\varepsilon_r \cong 1.75$ (**Supplementary Figure 9**), resulting in $\varepsilon = \varepsilon_0\ \varepsilon_r = 1.57 \times 10^{-11}\ \frac{F}{m}$.

width (mm)	length (mm)	area (mm^2)	distance (mm)	C (pF)
10	60	600	0.8	11.75
15	60	900	0.8	17.63
10	70	700	0.8	13.71
15	70	1050	0.8	20.57
10	80	800	0.8	15.67
15	80	1200	0.8	23.51

Supplementary Table 5. Values for the serial resistance and parasitic inductance of the connection lines used in the simulations.

	R_{ser} (Ω)	L_{par} (µH)
$Line_1$ (to C_1)	2.1	0.286
$Line_2$ (to C_2)	1.1	0.104

Supplementary Table 6. Coupling coefficients calculated from **Equation 8**. r_1 represents the effective radius of the textile inductor (arithmetic mean between inner and outer radii) and r_2 represents the effective radius of the reader inductor (**Equation 9**).

Sub-inductor	r_{in} (mm)	r_{out} (mm)	r_1 (mm)	r_2 (mm)	d (mm)	k
L_1	19	20	19.5	20	1	0.352
	19	20	19.5	20	2	0.349
	19	20	19.5	20	5	0.328
	19	20	19.5	20	10	0.266
L_2	17	18	17.5	20	1	0.352
	17	18	17.5	20	2	0.349
	17	18	17.5	20	5	0.325
	17	18	17.5	20	10	0.260
L_3	15	16	15.5	20	1	0.352
	15	16	15.5	20	2	0.348
	15	16	15.5	20	5	0.323
	15	16	15.5	20	10	0.254

Supplementary Table 7. Predicted resonance frequencies from LT spice simulations with all possible values for C_1 and C_2 (Supplementary Table 3) only for $C_1 > C_2$. The shaded grey areas indicate combinations for which the resonance frequencies are less than 20 MHz apart from each other and therefore such combinations were excluded.

C_1 (pF)	C_2 (pF)	f_{res1} (MHz)	f_{res2} (MHz)	$f_{res2} - f_{res1}$ (MHz)
13	12	46.77	70.79	24.02
14	12	45.71	70.79	25.08
14	13	45.71	67.61	21.90
15	12	43.65	70.79	27.14
15	13	43.65	67.61	23.96
15	14	43.65	64.57	20.92
16	12	42.66	70.79	28.13
16	13	42.66	67.61	24.95
16	14	42.66	64.57	21.91
16	15	42.66	63.10	20.44
17	12	41.69	70.79	29.1
17	13	41.69	67.61	25.92
17	14	41.69	64.57	22.88
17	15	41.69	63.1	21.41
17	16	41.69	61.66	19.97
18	12	39.81	70.79	30.98
18	13	39.81	67.61	27.8
18	14	39.81	64.57	24.76
18	15	39.81	63.10	23.29

Continued

18	16	39.81	61.66	21.85
18	17	39.81	58.88	19.07
19	12	38.9	70.79	31.89
19	13	38.9	67.61	28.71
19	14	38.9	64.57	25.67
19	15	38.9	63.10	24.2
19	16	38.9	61.66	22.76
19	17	38.9	58.88	19.98
19	18	38.9	57.54	18.64
20	12	38.02	70.79	32.77
20	13	38.02	67.61	29.59
20	14	38.02	64.57	26.55
20	15	38.02	63.1	25.08
20	16	38.02	61.66	23.64
20	17	38.02	58.88	20.86
20	18	38.02	57.54	19.52
20	19	38.02	56.23	18.21
21	12	37.15	70.79	33.64
21	13	37.15	67.61	30.46
21	14	37.15	64.57	27.42
21	15	37.15	63.10	25.95
21	16	37.15	61.66	24.51
21	17	37.15	58.88	21.73
21	18	37.15	57.54	20.39
21	19	37.15	56.23	19.08
21	20	37.15	54.95	17.8
22	12	36.31	70.79	34.48
22	13	36.31	67.61	31.30
22	14	36.31	64.57	28.26
22	15	36.31	63.1	26.79
22	16	36.31	61.66	25.35
22	17	36.31	58.88	22.57
22	18	36.31	57.54	21.23
22	19	36.31	56.23	19.92
22	20	36.31	54.95	18.64
22	21	36.31	53.7	17.39
23	12	35.48	70.79	35.31
23	13	35.48	67.61	32.13
23	14	35.48	64.57	29.09
23	15	35.48	63.10	27.62
23	16	35.48	61.66	26.18
23	17	35.48	58.88	23.4

Continued

23	18	35.48	57.54	22.06
23	19	35.48	56.23	20.75
23	20	35.48	54.95	19.47
23	21	35.48	53.7	18.22
23	22	35.48	52.48	17
24	12	34.67	70.79	36.12
24	13	34.67	67.61	32.94
24	14	34.67	64.57	29.9
24	15	34.67	63.10	28.43
24	16	34.67	61.66	26.99
24	17	34.67	58.88	24.21
24	18	34.67	57.54	22.87
24	19	34.67	56.23	21.56
24	20	34.67	54.95	20.28
24	21	34.67	53.7	19.03
24	22	34.67	52.48	17.81
24	23	34.67	51.29	16.62

Supplementary Table 8. Parameter space for each classification model used in the hyperparameter tuning steps. For all algorithms the random state was set to 42.

Classification algorithm	Parameters	Ranges / values
K-Nearest Neighbors	n_neighbors	range(1, 20)
	weights	uniform, distance
	algorithm	auto, brute
Logistic Regression	c	0.01, 0.1, 1, 10, 100
	penalty	l1, l2
	solver	lbfgs
	max_iter	5000, 10000
	class_weight	balanced
Support Vector Machine	c	0.01, 0.1, 1, 10, 100
	kernel	linear, rbf, poly
	gamma	scale, auto, 0.01, 0.1, 1
	degree	2, 3, 4
	class_weight	balanced
Decision Trees	criterion	log_loss, entropy, gini
	max_depth	2, 4, 6
	min_samples_leaf	3, 5, 7
	min_samples_split	8, 10, 12
	class_weight	balanced
Random Forest	max_depth	2, 4, 6
	n_estimators	10, 20, 50
	min_samples_leaf	1, 2, 5
	min_samples_split	2, 5, 7, 10
	class_weight	balanced
Extreme Gradient Boosting	n_estimators	100, 200
	learning_rate	0.01, 0.1, 0.2
	max_depth	2, 4, 6
	subsample	0.5, 0.9

Supplementary Table 9. Resonance frequency shifts for the bench tests and corresponding simulation results ($\Delta f_{res,1}$ refers to the resonance shift for sub-circuit 1 with capacitive sensor C_1). The percentage difference between simulations

Strain	Measurement		Simulation		Difference %	
	$\Delta f_{res,1}$ (MHz)	$\Delta f_{res,2}$ (MHz)	$\Delta f_{res,1}$ (MHz)	$\Delta f_{res,2}$ (MHz)	Sensor 1	Sensor 2
0.05	1.44	1.79	1.00	2.20	36	21
0.10	2.43	3.32	1.80	3.50	30	5
0.15	3.46	5.09	2.47	4.56	33	11
0.20	4.29	6.64	3.50	6.02	20	10

References

Bello, H., Zhou, B., Suh, S., and Lukowicz, P. (2021). MoCapaci: Posture and gesture detection in loose garments using textile cables as capacitive antennas., in *2021 International Symposium on Wearable Computers*, (New York, NY, USA: ACM). doi: 10.1145/3460421.3480418

Cheng, J., Amft, O., and Lukowicz, P. (2010). “Active capacitive sensing: Exploring a new wearable sensing modality for activity recognition,” in *Lecture Notes in Computer Science*, (Berlin, Heidelberg: Springer Berlin Heidelberg), 319–336.

Galli, V., Ahmadizadeh, C., Kunz, R., and Menon, C. (2023). Textile-based body capacitive sensing for knee angle monitoring. *Sensors (Basel)* 23, 9657.

Jin, Y., Glover, C. M., Cho, H., Araromi, O. A., Graule, M. A., Li, N., et al. (2020). Soft sensing shirt for shoulder kinematics estimation., in *2020 IEEE International Conference on Robotics and Automation (ICRA)*, (IEEE). doi: 10.1109/icra40945.2020.9196586

Kasap, D., Najafi, T. A., Thevenot, J. P. R., Dan, J., Albini, S., and Atienza, D. (2025). VersaPants: A loose-fitting textile capacitive sensing system for lower-body motion capture. *arXiv [eess.SP]*. doi: 10.48550/arXiv.2511.16346

Ma, D., Wu, Q., Fang, H., Tao, X., Shi, S., Wu, F., et al. (2024). Skin-core-fiber-based fabric integrated with pressure sensing and deep learning for posture recognition. *Nano Energy* 132, 110376.

Milovic, M., Farías, G., Fingerhuth, S., Pizarro, F., Hermosilla, G., and Yunge, D. (2022). Detection of human gait phases using textile pressure sensors: A low cost and pervasive approach. *Sensors (Basel)* 22, 2825.

Mokhlespour Esfahani, M. I., and Nussbaum, M. A. (2019). Classifying diverse physical activities using “smart garments.” *Sensors (Basel)* 19, 3133.

Tavassolian, M., Cuthbert, T. J., Napier, C., Peng, J., and Menon, C. (2020). Textile-based inductive soft strain sensors for fast frequency movement and their application in wearable devices measuring multiaxial hip joint angles during running. *Adv. Intell. Syst.*, 1900165.

Vu, C. C., and Kim, J. (2018). Human motion recognition by textile sensors based on machine learning algorithms. *Sensors (Basel)* 18, 3109.

Vu, C. C., and Kim, J. (2020). Highly elastic capacitive pressure sensor based on smart textiles for full-range human motion monitoring. *Sens. Actuators A Phys.* 314, 112029.

Xu, G., Wan, Q., Deng, W., Guo, T., and Cheng, J. (2022). Smart-Sleeve: A wearable textile pressure sensor array for human activity recognition. *Sensors (Basel)* 22, 1702.

Yu, T. C., Zhang, M. M., He, P., Lee, C.-J., Cheesman, C., Mahmud, S., et al. (2024). SeamPose: Repurposing seams as capacitive sensors in a shirt for upper-body pose tracking., in *Proceedings of the 37th Annual ACM Symposium on User Interface Software and Technology*, (New York, NY, USA: ACM), 1–13.

Zhang, W., Tashakori, A., Jiang, Z., Servati, A., Narayana, H., Soltanian, S., et al. (2023). Intelligent Knee Sleeves: A real-time multimodal dataset for 3D lower body motion estimation using smart textile. *arXiv [cs.CV]*. Available at: http://arxiv.org/abs/2311.12829

Zhou, B., Geissler, D., Faulhaber, M., Gleiss, C. E., Zahn, E. F., Ray, L. S. S., et al. (2023). MoCaPose: Motion capturing with textile-integrated capacitive sensors in loose-fitting smart garments. *Proc. ACM Interact. Mob. Wearable Ubiquitous Technol.* 7, 1–40.
